# Direct Quantitation of Phytocannabinoids by One-Dimensional ^1^H qNMR and Two-Dimensional ^1^H-^1^H COSY qNMR in Complex Natural Mixtures

**DOI:** 10.3390/molecules27092965

**Published:** 2022-05-05

**Authors:** Evangelos Dadiotis, Vangelis Mitsis, Eleni Melliou, Prokopios Magiatis

**Affiliations:** 1 Laboratory of Pharmacognosy and Natural Products Chemistry, Department of Pharmacy, National and Kapodistrian University of Athens, Panepistimiopolis Zografou, 15771 Athens, Greece; vaggdad@gmail.com (E.D.); emelliou@pharm.uoa.gr (E.M.); 2 Ekati Alchemy Lab SL, Carretera Barcelona 11, 08180 Moia, Spain; ekatimed@gmail.com

**Keywords:** cannabinoids, cannabidiol (CBD), cannabinol (CBN), cannabigerol (CBG), tetrahydrocannabinol (THC), cannabichromene (CBC), cannabielsoin (CBE), nuclear magnetic resonance spectroscopy (NMR), COSY NMR, qNMR

## Abstract

The widespread use of phytocannabinoids or cannabis extracts as ingredients in numerous types of products, in combination with the legal restrictions on THC content, has created a need for the development of new, rapid, and universal analytical methods for their quantitation that ideally could be applied without separation and standards. Based on previously described qNMR studies, we developed an expanded ^1^H qNMR method and a novel 2D-COSY qNMR method for the rapid quantitation of ten major phytocannabinoids in cannabis plant extracts and cannabis-based products. The ^1^H qNMR method was successfully developed for the quantitation of cannabidiol (CBD), cannabidiolic acid (CBDA), cannabinol (CBN), cannabichromene (CBC), cannabichromenic acid (CBCA), cannabigerol (CBG), cannabigerolic acid (CBGA), Δ9-tetrahydrocannabinol (Δ9-THC), Δ9-tetrahydrocannabinolic acid (Δ9-THCA), Δ8-tetrahydrocannabinol (Δ8-THC), cannabielsoin (CBE), and cannabidivarin (CBDV). Moreover, cannabidivarinic acid (CBDVA) and Δ9-tetrahydrocannabivarinic acid (Δ9-THCVA) can be distinguished from CBDA and Δ9-THCA respectively, while cannabigerovarin (CBGV) and Δ8-tetrahydrocannabivarin (Δ8-THCV) present the same ^1^H-spectra as CBG and Δ8-THC, respectively. The COSY qNMR method was applied for the quantitation of CBD, CBDA, CBN, CBG/CBGA, and THC/THCA. The two methods were applied for the analysis of hemp plants; cannabis extracts; edible cannabis medium-chain triglycerides (MCT); and hemp seed oils and cosmetic products with cannabinoids. The ^1^H-NMR method does not require the use of reference compounds, and it requires only a short time for analysis. However, complex extracts in ^1^H-NMR may have a lot of signals, and quantitation with this method is often hampered by peak overlap, with 2D NMR providing a solution to this obstacle. The most important advantage of the COSY NMR quantitation method was the determination of the legality of cannabis plants, extracts, and edible oils based on their THC/THCA content, particularly in the cases of some samples for which the determination of THC/THCA content by ^1^H qNMR was not feasible.

## 1. Introduction

*Cannabis sativa* is a plant with a very long history. Over the centuries, cannabis was cultivated mainly for its fibres and also for medicinal, recreational, and religious purposes. In 1925 and 1937, cannabis was regulated as a dangerous drug in both Europe and the US, respectively. However, for the past 50 years, an increasingly intense political and scientific debate has surrounded the legalization of the plant and the safety issues of cannabis-based medical treatments. Today, dozens of cannabis-based products are available on the market, and various formulations of cannabis ingredients have been approved by drug organizations [1]. As a result of the increasing growth of medicinal, nutritional, and recreational cannabis preparations, there is a growing demand for the development of qualitative and quantitative methods for the analysis of the bioactive components of cannabis.

In recent years, more than 500 substances have been isolated from the plant *Cannabis sativa,* of which more than 150 belong to the category of cannabinoids. The basic structure of these substances is terpenophenolic with 21 carbon atoms, and it is chemically related to terpenes with their ring structure derived from a geranyl pyrophosphate (C10 monoterpene subunit), but derivatives and transformation products also belong to the category of cannabinoids. These compounds can be classified into 11 different types: Δ9-trans-tetrahydrocannabinol (Δ9-THC), Δ8-trans-tetrahydrocannabinol (Δ8-THC), cannabigerol (CBG), cannabichromene (CBC), cannabidiol (CBD), cannabinodiol (CBND), cannabielsoin (CBE), cannabicyclol (CBL), cannabinol (CBN), cannabitriol (CBT), and miscellaneous types [2,3].

Quantitative analysis of cannabinoids is performed primarily using high performance liquid chromatography combined with ultraviolet detector or mass detector (HPLC–UV/MS) and gas chromatography combined with flame detector or mass detector (GC–FID/MS) [4,5,6,7,8,9,10,11,12,13,14,15,16,17,18,19,20,21]. These methods are well-established and quite effective in the field of cannabis analysis. However, several studies have shown that the chromatographic separation of cannabinoids and their quantitation often present difficulties. For example, chromatography may not provide enough resolution to analyse complex cannabis mixtures [22,23,24]. The quantitation of cannabinoids via GC analysis is not suitable for measuring acidic cannabinoids the analysis of acidic cannabinoids with GC requires an extra derivatization step [9,23,25]; and liquid chromatography is a highly solvent-consuming technique. In addition, the afore-mentioned methods usually require the samples to be treated for purification from impurities, such as chlorophylls and lipids, prior to analysis [26,27]. In addition, these methods require the preparation of calibration curves at regular intervals, and some of the cannabinoid standards are illegal, very expensive, and/or unavailable.

Quantitation with nuclear magnetic resonance spectroscopy (qNMR) seems to overcome the afore-mentioned difficulties and is widely used in the analysis of natural products [28,29,30,31,32]. Additionally, NMR methods have been used specifically for the quantitation of cannabinoids with quite acceptable levels of repeatability and reproducibility and a very short analysis time for both ^1^H and ^13^C qNMR methods [26,27,33,34,35,36]. In the current work, we investigated the expansion of a previously described qNMR method [35] to a wider range of 16 cannabinoids and the application of the new method to various samples. However, during the development and application of the ^1^H qNMR method, we observed that certain characteristic peaks used for quantitation presented overlaps, thus preventing accurate measurements. To overcome this problem, we developed an alternative quantitation method based on 2D-COSY qNMR, which we then used for 7 cannabinoids: cannabigerol, cannabigerolic acid, cannabidiol, cannabidiolic acid, Δ9tetrahydrocannabinol, Δ9-tetarahydrocannabinolic acid. The two methods were applied to a wide range of *Cannabis* plant samples and products. Specifically, the methods were applied in the analysis of hemp plants; cannabis extracts; edible cannabis, coconut, and hemp seed oils; and cosmetic products containing cannabinoids.

## 2. Results

### 2.1. ^1^H-NMR Peak Assignment

**Figure 1:**
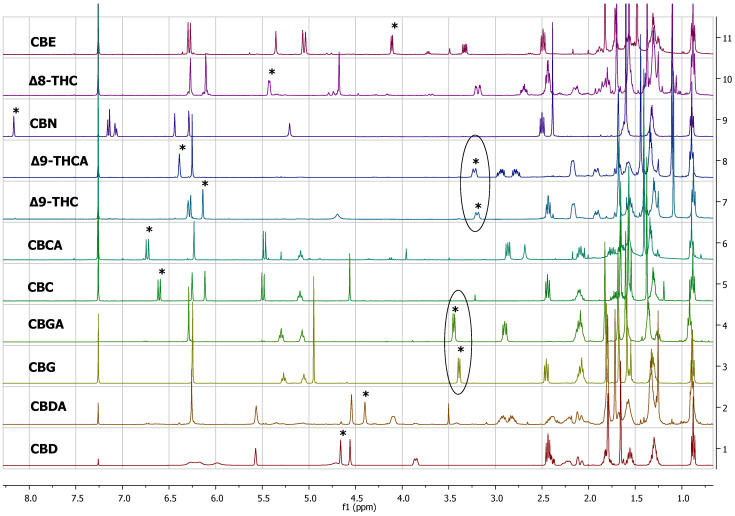
^1^H-NMR spectra of pure cannabinoids CBD, CBDA, CBG, CBGA, CBC, CBCA, Δ9-THC, Δ9-THCA, CBN, Δ8-THC, and CBE dissolved in CDCl_3_. Selected signals of Table 1 are marked with asterisks.

The peaks that could be used for quantitation of each cannabinoid are presented in Table 1. More specifically, for the quantitation of CBD, the peak at 4.66 ppm was used, for which there was not any overlap with other peaks in any plant material or cannabis-based product that was analysed. Similarly, for the quantitation of CBDA, the peak at 4.40 ppm was used. For the quantitation of CBC and CBCA, the peaks at 6.61 ppm and 6.73 ppm were used, respectively. For Δ8-THC, the most characteristic peak was at 5.43 ppm, and for CBN, 8.18 ppm. For the quantitation of Δ9-THC, the peaks at 3.21 ppm and 6.14 ppm could be used, and for the quantitation of Δ9-THCA, the peaks at 6.40 ppm and 3.24 ppm were used. For CBE, the peak at 4.11 ppm could be distinguished from other peaks (Table 1, Figure 1). In contrast to the previous cases, the ^1^H-NMR spectrum of CBG was very similar with that of CBGA, showing only a slight difference between the H-1 doublet peak at 3.41 ppm and 3.45 ppm, respectively, which was observable only when the spectrum was recorded for the pure substances. However, in some complex mixtures, CBG and CBGA were present together, along with other cannabinoids and other substances from the cannabis plant, and in that case, these two peaks overlapped, such that it was difficult in some samples to quantify these two compounds separately.

### 2.2. Cannabinoid Varins

**Figure 2:**
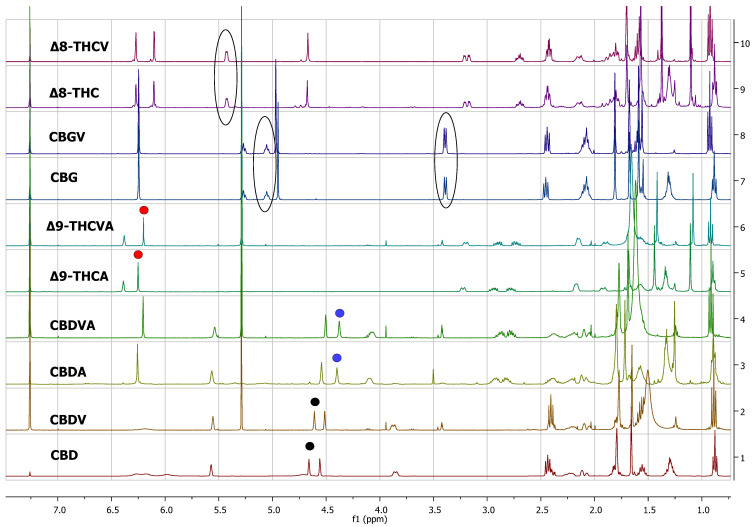
^1^H-NMR spectra of pure cannabinoids CBD, CBDV, CBDA, CBDVA, Δ9-THCA, Δ9-THCVA CBG, CBGV, Δ8-THC, and Δ8-THVC dissolved in CDCl_3_. The selected signals of CBD and CBDV are marked with black dots, the selected signals of CBDA and CBDVA are marked with blue dots, and the selected signals of THCA and THCVA are marked with red dots. The encircled peaks highlight the similarities between CBG and CBGV and between Δ8-THC and Δ8-THCV.

The proton NMR spectra of cannabinoid varins were also recorded. Cannabinoid varins can easily be separated from each other; however, separation from major cannabinoids involved several difficulties. CBGV has a spectrum similar to that of CBG, with the only difference being the lack of a multiplet peak at 1.29–1.34 ppm. The same problem was noticed in Δ8-THCV and Δ8-ΤHC ^1^H-NMR spectra. Therefore, the integration of the signals resulted in the sum of CBG with CBGV and Δ8-THC with Δ8-THCV, respectively. However, the peaks of CBDV, CBDVA, and THCVA could be separated from those of CBD, CBDA, and THCA in proton NMR spectra, respectively. The H-10 *cis* peak of CBDV was located at 4.52 ppm, while that of the H-10 *trans* was at 4.61 ppm; the CBD H-10 *cis* peak was located at 4.56 ppm, while the H-10 *trans* peak was at 4.66 ppm. CBDV could also be distinguished from all of the other cannabinoids included in this study. The H-10 *cis* peak of CBDVA was located at 4.50 ppm, and the H-10 *trans* peak was at 4.38 ppm; The CBDA H-10 *cis* peak was at 4.40 ppm, and H-10 *trans* peak was at 4.56 ppm. The H-4 peak of Δ9-THCVA was at 6.20 ppm, and the Δ9-THCA H-4 peak was at 6.28 ppm (Table 2, Figure 2).

### 2.3. ^1^H-^1^H COSY qNMR

#### 2.3.1. The ^1^H-^1^H COSY qNMR Method

In some complex mixtures, the quantitation of some cannabinoids using qHNMR was not possible due to overlapping peaks. To overcome this problem, we developed a novel 2D qNMR method based on ^1^H-^1^H COSY NMR spectroscopy, in which the correlations of the peaks of the neighbouring protons of a compound are unfolded in two dimensions. Therefore, the resolution is increased with the added dimension as peaks are spread along an additional orthogonal dimension. However, the direct quantitative properties observed in the ^1^H-NMR spectra cannot be extrapolated to 2D NMR. Cross-peak intensities are influenced by other factors apart from concentration, such as relaxation times, mixing times, evolution times, uneven magnetization transfers, and coupling constants [37]. It was therefore necessary to prepare appropriate calibration curves for each analysed compound. It is important to note here that, unlike chromatographic quantitation methods in which the calibration curves must be repeated frequently, in 2D qNMR they must be made only once.

#### 2.3.2. ^1^H-^1^H COSY NMR Spectrum Peak Correlations Assignment

**Figure 3:**
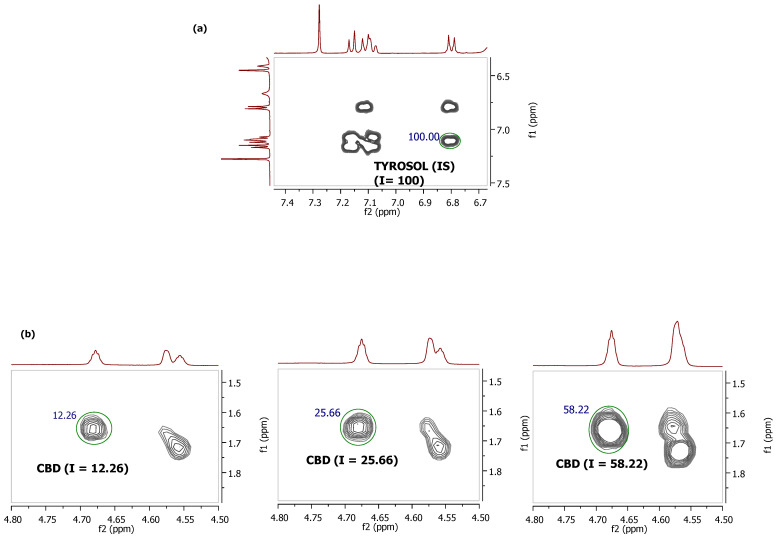
Cannabidiol integration in COSY NMR for calibration curve construction: (**a**) The integration of tyrosol (IS) cross peak (7.11 ppm/6.80 ppm) was set as 100; (**b**) The integrations of CBD cross peak (4.68 ppm/1.65 ppm) in various increasing concentrations of 1 mg/0.75 mL, 2 mg/0.75 mL, and 5 mg/0.75 mL, with stable IS concentration (1 mg/0.75 mL) are 12.26, 25.66, and 58.22, respectively.

The peaks that could be used for each cannabinoid are shown in Table 3. The range of integration for each peak is presented in the Supplementary Material in Table S1. Tyrosol was selected as an internal standard because it has a strong signal at 7.11 ppm/6.80 ppm, it does not overlap with cannabinoid peaks or those of any other substance of the cannabis plant, and in addition, it is a stable compound that is not present in the cannabis plant. The calibration curves for each cannabinoid that was quantitated with the COSY NMR method were constructed by making a mixture of the reference standards at various concentrations with the internal standard. The correlations of the peaks that were used for the quantitation of each cannabinoid were chosen based on the intensity of the signals to achieve the minimum LOD and LOQ. CBD was quantitated using the cross peak at 4.66 ppm/1.66 ppm and CBDA with the cross peak at 4.56 ppm/4.40 ppm. CBG and CBGA were measured as a whole (5.31 ppm/3.42 ppm), because it was difficult to separate their signals at the COSY NMR spectrum, and also to increase the sensitivity of this method. The same was applied for the quantitation of THC and THCA (3.25 ppm and 1.68 ppm). Different ratios of concentrations of the acidic and neutral forms of CBG/CBGA and THC/THCA were made to determine whether the presence or absence of the acidic group affects the intensity and the integration of the peaks in the COSY NMR spectrum. No significant difference between the acidic and the neutral forms were found, permitting the integration of the cross peaks as a whole. For the quantitation of CBN, the signal at 8.18 ppm/7.07 ppm was used. In Table 3, the linearity of the constructed calibration curves is also presented. Figure 3 provides an example of the integration of the cross peaks for the construction of the calibration curve of CBD. As described in detail in Section 4.3.2, we prepared solutions with various known concentrations of CBD and a constant amount of the internal standard, then we integrated the cross-peak signal of Tyrosol (which was arbitrarily set as 100), and finally we integrated the selected signal of CBD. Based on the integration of the CBD cross peak and the known amount of this substance in the NMR tube, we prepared the calibration curve. The same technique was applied for the construction of the calibration curves of each cannabinoid.

### 2.4. Chemical Analysis

**Figure 4:**
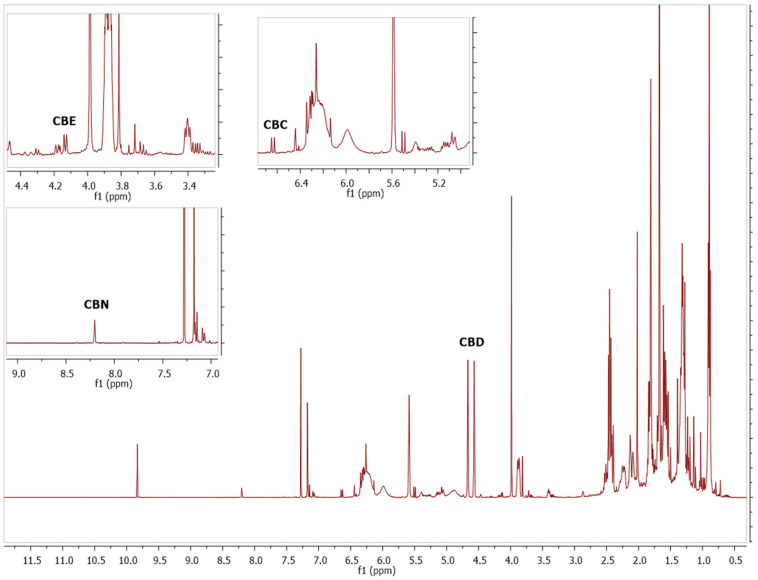
^1^H-NMR spectrum of hemp extract after distillation.

**Figure 5:**
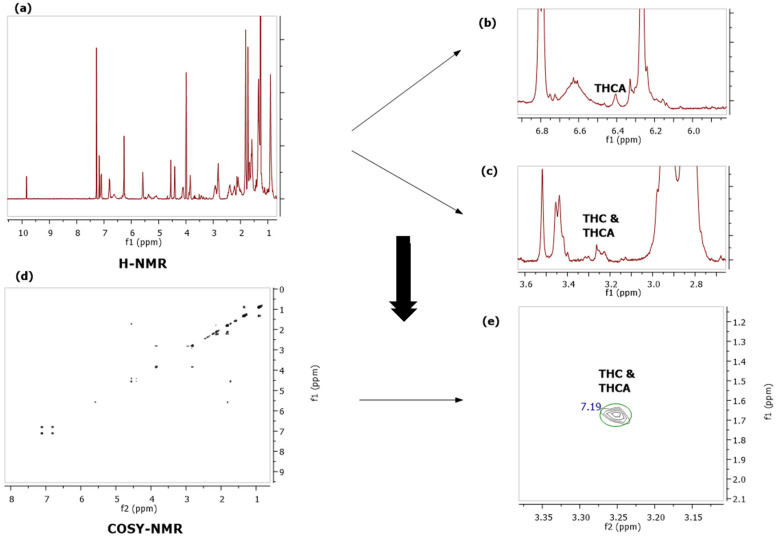
Δ9-THC quantitation with ^1^H-^1^H-COSY qNMR in plant material: (**a**) ^1^H-NMR spectrum of hemp plant; (**b**,**c**) detection of the presence of Δ9-THC in the material; and (**d**,**e**) Integration of the selected cross peak and quantitation of Δ9-THC & Δ9-THCA with ^1^H-^1^H COSY qNMR.

The developed methods were applied for the screening and quantitation of cannabinoids in plant material, cannabis extracts, edible cannabis oils, and cannabis-based cosmetic products. Depending on the concentration of the cannabinoids in each material, a different portion of the starting material should be used for the analysis. CBD, CBDA, CBG, and CBGA are the major cannabinoids in hemp varieties and are also the major cannabinoids in cannabinoid edible oils and cannabis-based cosmetic products. CBN is not usually present in industrial cannabis samples, and it can only be found in very low concentrations in hemp-based material. CBC and CBCA are usually around 0.5% in most of the industrial cannabis varieties, so it is important to measure this substance in cannabis plants, extracts, products, etc. CBE is a degradation product of CBD [38], and it is especially present in cannabis extracts after distillation (Figure 4). In addition, cannabicyclol (CBL) is a degradation product of CBC and is present in cannabis extracts and products that are exposed to heat and light [39]. Although it was difficult to quantify CBL with ^1^H-NMR, preliminary experiments showed that it could be distinguished using COSY NMR (data not presented). Δ8-THC is not usually present in hemp or hemp-based products although, nowadays, it is present in many products on the market, especially in the US. Δ9-THC and Δ9-THCA are usually found in low concentrations although the precise quantitation of these substances is crucial due to the legal framework. The developed methods were applied to plant materials that were required to be tested for their legality because the legal framework in Europe allows for the presence of up to 0.2% THC in industrial cannabis plants. In the following example, we observed that all THC and THCA peaks overlapped in the proton spectrum, and this obstacle was overcome with the COSY NMR spectrum, where the 3.25 and 1.69 ppm cross peak was distinguished from the other signals (Figure 5). As shown in Figure 5, with the ^1^H-NMR the presence of Δ9-THC was detected; however, due to the overlaps, it was not possible quantitate it, while in the COSY NMR spectrum the correlations of the peaks could be easily distinguished.

### 2.5. Comparison of the Two Methods

The developed COSY NMR method was applied in combination with the ^1^H-NMR quantitation method for the analysis of hemp plants as a tool to find the appropriate time period time for their harvest. More specifically, the in-plant content of CBD, CBDA, CBG, and CBGA, which are present in abundance in specific varieties of hemp plants, was measured. Additionally, the in-plant content of THC and THCA was measured, to determine whether the plants were within the legal framework (having THC and THCA contents of less than 0.2% of the dry weight of the plant material). Table 4 shows three examples of quantitation with COSY NMR compared to the results of quantitation with ^1^H-NMR (from an average of three measurements made for each experiment), where the percentage relative difference between the results of the two methods was less than 8%. (More details are given in Table S5.) These methods were also compared with HPLC–UV and GC–MS analysis for CBD, CBG, and CBN in a decarboxylated cannabis extract and showed less than 8% relative difference. More specifically, the relative difference between the COSY NMR and ^1^H-NMR, HPLC–UV, and GC–MS methods was from 1.71% to 7.12%, and the relative difference between the ^1^H-NMR and HPLC–UV, and GC–MS methods was from 0.41% to 4.84%, showing that all methods can give comparable results (See Supplementary Material Table S2).

### 2.6. Method Validation

The intraday precision, expressed as the relative standard deviation (RSD), ranged from 2.1 to 5.4% for CBD, CBDA, CBG, CBGA, THC, and THCA using the ^1^H-NMR and COSY NMR methods. The inter-day precision ranged between 2.9 and 6.3%, in both methods, respectively. The RSD values are adequate and indicate the suitability of the methods. The results for the accuracy were between 0.3 and 4.5% and are expressed as the relative percentage error (Er %), for both methods. The estimated accuracy values with the proposed method are within acceptable levels for the six analytes. The obtained data indicate that the method could be considered as accurate. The recoveries were found to be more than 90% for the 1 mg/100 mg levels for each cannabinoid in edible oils and cosmetic products, indicating acceptable recovery. The sensitivity of the method was represented by its LOD and LOQ, which were found to be from 0.015 to 0.04 mg/mL and 0.03 to 0.11 mg/mL, respectively, for each cannabinoid for the ^1^H-NMR method from 0.09 to 0.14 mg/mL and 0.17 to 0.22 mg/mL, respectively, for each cannabinoid for the ^1^H-^1^H COSY NMR method (More details can be found in Supplementary Material, Tables S3 and S4).

## 3. Discussion

NMR is an already-known, reliable, and powerful technique for the quantitation of natural products and cannabinoids present in the cannabis plant [26,27,33,34,35,36]. The main advantages of qNMR are its accuracy, reproducibility, and flexibility with respect to the nature of the analyte, the only requirement being the presence of protons and carbons, and its ability to simultaneously quantitate multiple analytes. Quantitative ^1^H-NMR has been shown to have an accuracy and precision level of ±1% and an uncertainty of measurement of less than 0.1%. In addition, NMR is non-destructive and does not require separation or derivatization, and it is amenable to compounds that are difficult to analyse by GC and LC techniques [40]. In qNMR spectroscopy, the experimental parameters can be adjusted in such a manner that the desired signal–to–noise ratio for detection and quantitation can be achieved [41]. Additionally, for this method, the use of standard substances and the construction of calibration curves is not necessary [42]. Due to the complexity of cannabis extracts, the overlapping of peaks may occur in ^1^H NMR, as may also happen in the HPLC and GC quantitation methods. With the COSY NMR method, the signals are spread over a broader area, and the risk of overlapping peaks is significantly lower. The COSY NMR method requires reference standards, which, in some cases, are difficult to find and which are also quite expensive. The advantage of the COSY NMR method, compared to LC and GC quantitation methods, is that the calibration curves must be made only once. In addition, the 1D and 2D NMR methods can be considered as supplementary to each other, and in less than 20 min, they can provide both qualitative and quantitative information on a complex cannabinoids mixture. The advantages of a combination of 1D and 2D quantitative NMR methods have been highlighted in recent publications [43,44]. Additionally, the concentrations of THC and THCA are measured with two different methods, and the probability of false results is reduced. Based on the literature, by using an NMR system with increased frequency the COSY NMR spectrum can be obtained in less than 5 min and provide quantitative results [45]. Preliminary results have shown that all cannabinoids mentioned in the ^1^H-NMR method, except CBGV and Δ8-THCV, can be quantitated with ^1^H-^1^H COSY NMR. Methods with UV and MS detectors appear to have problems due to the presence of artefacts, as well as to different absorption and ionization of each compound. NMR and FID are the methods that are the most independent detection of external factors although, with the use of FID, only the neutral forms of cannabinoids can be quantified. The European medical cannabis market lacks a common regulatory framework; as a result, there is a pressing need to develop clear policies and regulations across territories to foster harmonisation.

## 4. Materials and Methods

### 4.1. Reagents and Cannabinoid Samples

All solvents were of analytical grade (Merck, Darmstadt, Germany). Syringaldehyde (99% purity, Sigma-Aldrich, Steinem, Germany) and Tyrosol (99% purity, Sigma-Aldrich, Steinem, Germany) were used as internal standards (IS). IS solution was prepared in acetonitrile at a concentration of 0.5 mg/mL and kept at 4 °C. The IS solution was left to reach room temperature before use. The quantitative determination of cannabinoids was performed using one- and two-dimensional NMR spectroscopy with a Bruker Avance DRX 400 MHz (National and Kapodistrian University of Athens, Greece) and with an Agilent/Varian Inova three-channel 400 MHz spectrometer (University of Alberta, Canada). The ^1^H-NMR spectra and ^1^H-^1^H COSY NMR spectra were processed using the MNova (Mestrelab Research). Crystalline cannabidiol (CBD, purity 99.6%), crystalline cannabigerol (CBG, purity 98.7%), Δ8-tetrahydrocannabinol (Δ8-THC, 98%), and Δ9-tetrahydrocannabinolic acid (Δ9-THCA) were provided by the Ekati Alchemy Lab (Spain). Cannabidiolic acid (CBDA, 98.5%), cannabigerolic acid (CBGA, 98.3%), and cannabichromenic acid (CBCA, 94.8%) were isolated from hemp cannabis plants (with Δ9-THCA < 0.2%, provided by the Hellenic Agricultural Organization “Demeter” (Athens). Δ9-tetrahydrocannabinol (Δ9-THC) and cannabichromene (CBC) occurred via the decarboxylation of Δ9-THCA and CBCA, respectively. Δ8-tetrahydrocannabivarin (Δ8-THCV, 99.1%) and cannabigerovarin (CBGV, 98.1%) were provided by Salzman Group, LTD (Israel). Cannabielsoin (CBE, 96%) was isolated from distillate CBD extract (Ekati Alchemy Lab, Spain). The cannabinoid varins ∆9-tetrahydrocannabivarinic acid (∆9-THCVA), cannabidivarin (CBDV), and cannabidivarinic acid (CBDVA) were purchased from Sigma-Aldrich, Germany, as reference standards in acetonitrile 1 mg/mL solution. All cannabis extracts, cannabis-based cosmetic products, and cannabis edible oils were provided by the Ekati Alchemy Lab (Spain). Plant material R1N135, R4N120, and R3N110 were provided from selected CBD and CBG varieties grown in the greenhouse at the Institute of Mediterranean and Forest Ecosystems of the Hellenic Agricultural Organization “Demeter”.

### 4.2. ^1^H-NMR Quantitation

^1^H-NMR quantitation of cannabinoids was performed based on the method that we described in a previous study [35]. Briefly, 100 mg of dry material, obtained after two heating and freeze-drying steps, were dispersed and sonicated in 10 mL of 9:1 methanol: chloroform volumetric ratios. After centrifugation, the clear supernatant with the internal standard was evaporated. The resulting matter was later dissolved in deuterated solvent for the NMR analysis and added to a 5 mm NMR tube. The specific detailed procedure for different substrates is described in Section 4.4.1, Section 4.4.2, Section 4.4.3 and Section 4.4.4 Syringaldehyde was used as an internal standard, which has not been reported to be present in cannabis extracts and which shows only three single peaks in the ^1^H-NMR spectrum, thus reducing the chances of overlapping proton peaks used to quantitate cannabinoids. Each sample was analysed with a standard 90-degree excitation pulse, with a pulse width of 10 μs and a prescan delay of 6.5 μs. All measurements were performed at 298 K. Typically, 16 scans were collected into 32 K data points over a spectral width of 0–13 ppm (5263.18 Hz) with a relaxation delay of 10 s, an acquisition time of 3.11 s, and an FID resolution of 0.32 Hz. The appropriate relaxation delay was determined by gradual increases (1, 2, 5, 8, 10, 15, 20 s) until the ratio between the integration of the peak of internal standard and the peak of the target compounds remained unchanged. (More details can be found in the Supplementary Material in Table S6.) The matching, tuning, shimming, and receiver gain adjustments, as well as phasing and baseline correction, were always first performed automatically and then manually to achieve the best results. Prior to Fourier transformation (FT), an exponential weighting factor corresponding to a line broadening of 0.3 Hz was applied. For the peaks of interest, accurate integration was performed manually. The concentration of cannabinoids was measured by comparing the area of the selected signal of Table 1 with that of the internal standard (IS) at 9.81 ppm, which was set as 1. The calculation of the concentration of each cannabinoid in mg/100 mg of dry material was performed using the following formula:

n_CB_ = n_is_ × a × I_CB_/I_is_ (1)

m_CB_ = n_CB_/MW_CB_ (2)

where n_is_ = 0.0027 mmol (Syringaldehyde MW = 182.17), I_is_ = 1, a = 1 (for one proton peaks) or 0.5 (for two protons peaks), n_CB_ are the moles of each cannabinoid, m_CB_ is the mass of each cannabinoid, and MW_CB_ is the molecular weight of each cannabinoid, as shown in Table 1.

### 4.3. ^1^H-^1^H COSY qNMR Method

#### 4.3.1. ^1^H-^1^H COSY qNMR Quantitation

The pulse program cosygpmfppqf was used, with a spectral width of 4854.369 Hz; dummy scan, 16; number of scans, 2; acquisition time was 0.21 s; 2K (F2) × 256 (F1) data points. Processing was as follows: Zero filling and FT to 1 K × 1 K data points after multiplication with sine filter. All NMR data were processed using MNova (Mestrelab Research). More specifically, the method was applied to quantitate CBD, CBDA, CBG, CBGA, CBN, THC, and THCA by making a calibration curve for each substance. Tyrosol (Sigma-Aldrich) was used as an internal standard, where the signal displaying at 6.80/7.11 ppm was used for integration. More details about the COSY NMR experiment can be found in Table S7 in the Supplementary Materials.

#### 4.3.2. Calibration Curves for ^1^H-^1^H COSY qNMR

Calibration curves were constructed for each cannabinoid. Matrix solutions with 10 mg/mL CBD, 9 mg/mL CBDA, 4 mg/mL CBG, 4 mg/mL CBGA, 8 mg/mL CBN, 6.8 mg Δ9-THCA, 1 mg/mL tyrosol, and 0.5 mg/mL syringaldehyde solution were initially prepared. Then, 5 different concentrations of each matrix solution and 1 mg/mL tyrosol and 0.5 mg/mL syringaldehyde solution were added to round-bottomed flasks. The final solutions were evaporated, and the mixtures were diluted in 750 μL CDCl_3_ and transferred into 5 mm NMR tubes. The ^1^H-NMR and COSY NMR spectra of the above-described solutions were then obtained.

### 4.4. Chemical Analysis

#### 4.4.1. Plant Extraction and Chemical Analysis of Plant Material

The extraction process of the plant material was based on the recommended extraction method of the *United Nations Manual* [46] where MeOH/CHCl_3_ (9: 1) was used as the solvent extraction system. More specifically, a quantity of plant material with the corresponding volume of solvent (1:10 g/mL), was extracted in an ultrasonic bath for 15 min, and every 5 min, the plant material was stirred for 10 s using a vortex. The samples were then centrifuged at 4000 rpm for 5 min, and the solvent was obtained. Finally, the solution was evaporated in vacuum to give a cannabinoid extract. All samples were dried at 60 °C till constant weight and were kept in a desiccator. The flower samples were milled and were placed overnight in a deep freezer (−76 °C). A second freeze-drying step took place for 12 h at −52 °C and 0.03 mbar pressure to ensure the almost-complete removal of moisture. Then, 100 mg (±0.1 mg) of the dried, ground flower sample was weighed and placed in 15 mL Falcon plastic tubes and 10 mL 90:10 methanol/chloroform mixture (Panreac) was added for analysis. The tubes were transferred to an ultrasonic bath (Semat, St. Albans, UK) for 15 min to complete the extraction. The samples were centrifuged at 3075× *g* for 5 min (Eppendorf 5810R, Hamburg, Germany). A quantity of 10 mL of the clear supernatant was carefully removed and transferred to 50 mL round-bottom flasks, where it were mixed with 1 mL of a syringaldehyde (Sigma-Aldrich) solution (0.5 mg/mL) in acetonitrile (Scharlau) (internal standard, IS) and with 1 mL of tyrosol (Sigma-Aldrich) solution (1 mg/mL) in methanol (Merck) (internal standard, IS) and the mixture was evaporated in a vacuum rotary evaporator (Buchi, Flawil, Switzerland). The extract of each sample obtained from the above-described procedure was dissolved in 750 μL deuterated chloroform (CDCl_3_) (Merck), the solution was transferred to a 5 mm NMR tube, and analysis was achieved as described above.

#### 4.4.2. Chemical Analysis of Cannabis Extracts

In a 50 mL, round-bottom flask, 20 mg of cannabis extract was weighed; subsequently, 1 mL of a syringaldehyde (Sigma-Aldrich) solution (0.5 mg/mL) in acetonitrile (Merck) (internal standard, IS) and 1 mL of Tyrosol (Sigma-Aldrich) solution (1 mg/mL) in methanol (Merck) (internal standard, IS) were added, and the mixture was homogenized and evaporated in a vacuum rotary evaporator (Buchi, Flawil, Switzerland). The extract and the internal standards were dissolved in 750 μL deuterated chloroform (CDCl_3_) (Merck), the solution was transferred to a 5 mm NMR tube, and analysis was performed as described above.

#### 4.4.3. Chemical Analysis of Cannabinoid Oils

Cannabinoid oil (0.5 g) was mixed with cyclohexane (2 mL) and acetonitrile (10 mL). The mixture was homogenized using a vortex mixer for 30 s and centrifuged at 4000 rpm for 5 min. A part of the acetonitrile phase (0.5–2 mL) was collected, mixed with 1.0 mL of a syringaldehyde solution (0.5 mg/mL) in acetonitrile and 1 mL of Tyrosol (Sigma-Aldrich) solution (1 mg/mL) in methanol (Merck) (internal standard, IS), and evaporated under reduced pressure using a rotary evaporator (Buchi, Flawil, Switzerland).

#### 4.4.4. Chemical Analysis of Cosmetic Products with Cannabinoids

In a 15 mL FALCON plastic tube, 200 mg of each product was added, followed by the addition of acetonitrile (10 mL). The mixture was homogenized using a vortex mixer for 60 s. The tubes were transferred to an ultrasonic bath (Semat, St. Albans, UK) for 15 min to complete the extraction. The samples were centrifuged at 3075× *g* for 5 min (Eppendorf 5810R, Hamburg, Germany). A quantity of 10 mL of the clear supernatant was carefully removed and transferred to 50 mL, round-bottom flasks, where it were mixed with 1 mL of a syringaldehyde (Sigma-Aldrich) solution (0.5 mg/mL) in acetonitrile (Merck) (internal standard, IS) and 1 mL of tyrosol (Sigma-Aldrich) solution (1 mg/mL) in methanol (Merck) (internal standard, IS), and the mixture was evaporated in a vacuum rotary evaporator (Buchi, Flawil, Switzerland). The extract of each sample obtained from the above-described procedure was dissolved in 750 μL deuterated chloroform (CDCl_3_) (Merck), the solution was transferred to a 5 mm NMR tube, and analysis performed as described above.

### 4.5. Method Validation

Precision (calculated as the relative standard deviation % (RSD %)), accuracy (evaluated as the relative percentage error % (Er %)), and sensitivity (evaluated as the limits of detection (LOD) and quantitation (LOQ)) were measured. Spiked cannabinoid samples in MCT oil were prepared to give concentrations of each cannabinoid at 2.5, 5, 10, and 20 mg/mL and were analysed for the determination of the linearity. The relationship of the integration ratio of the analytes versus the IS and the corresponding concentration of the spiked cannabinoid standards was determined by unweighted linear regression analysis. The intraday precision was determined by analysing 5 replicates of spiked cannabinoid samples at 2 concentration levels (5 and 10 mg/mL MCT oil). The inter-day precision was assessed by analysing spiked cannabinoid samples at 2 concentration levels, namely 5 and 10 mg/mL levels, prepared on 5 different days. Spiked cannabinoid samples at 3 concentration levels, 5, 10, and 20 mg/mL, were analysed to determine the accuracy of the method. The results were expressed as the relative percentage error (Er %), defined as [assayed concentration − nominal concentration]/[nominal concentration] × 100. For the calculation of the recovery, spiked cannabinoid samples with concentrations of each cannabinoid at the 10 mg/mL level (n = 5 for each analyte) in MCT oil were analysed by employing the proposed extraction procedure. The recovery was calculated as the ratio of the response of both compounds in the spiked cannabinoid samples against that of the standards at the same levels and was expressed as the mean ± standard deviation (SD). The LOD and LOQ were determined by running six blank samples of each carrier of cannabinoids and measuring the background response at the chemical shift of each analyte. Signal–to–noise (S/N) ratios of 3:1 and 10:1 were used for the calculation of the LOD and LOQ, respectively. These methods were also compared with HPLC–UV and GC–MS analysis for CBD, CBG, and CBN. Details about these methods can be found in the Supplementary Materials.

## Figures and Tables

**Table 1:** Structures and selected signals (ppm) of cannabinoids which can be used for quantitation, depending upon the sample.

Cannabinoid	Structure	Proton	δ (ppm)
CBD	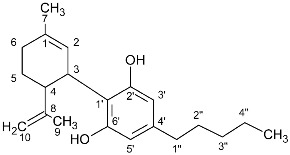	**H-10 *trans*** H-10 *cis* H-2	**4.66** 4.56 5.56
CBDA	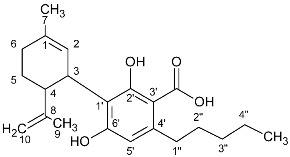	H-5′ H-10 *trans* **H-10 *cis*** H-2	6.27 4.56 **4.40** 5.56
CBG	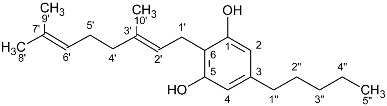	H-2/H-4 **H-1′a/H-1′b**	6.26 **3.41**
CBGA	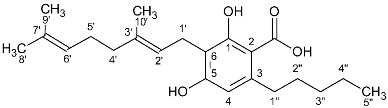	H-4 **H-1′a/H-1′b**	6.29 **3.45**
CBC	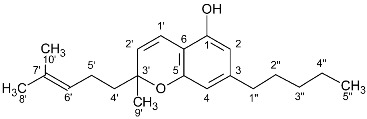	**H-1′** H-2′	**6.61** 5.50
CBCA	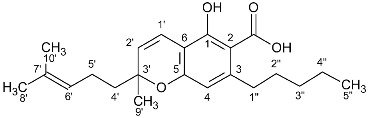	**H-1′** H-2′	**6.73** 5.48
CBN	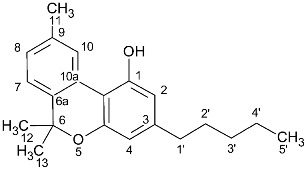	H-4 **H-10**	6.44 **8.18**
Δ8-THC	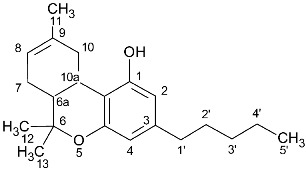	H-2 H-4 **H-8** H-10a	6.11 6.27 **5.43** 3.19
Δ9-THC	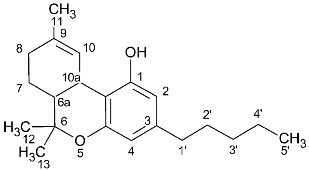	**H-2** H-4 H-10 **H-10a**	**6.14** 6.28 6.32 **3.21**
Δ9-THCA	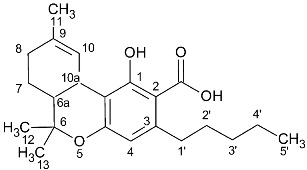	H-4 **H-10** **H-10a**	6.28 **6.40** **3.24**
CBE	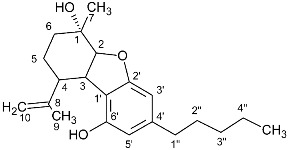	**H-2** H-3′ H-5′	**4.11** 6.30 6.27

**Table 2:** Structures and selected signals (ppm) of cannabinoid varins, which can be used for quantitation, depending on the sample.

Cannabinoid	Structure	Proton	δ (ppm)
CBDV	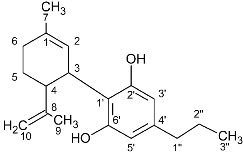	**H-10 *trans*** H-10 *cis*	**4.61** 4.52
CBDVA	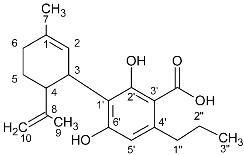	H-10 *trans* **H-10 *cis***	4.50 **4.38**
Δ9-THCVA	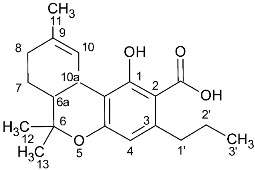	**H-4** H-10a	**6.20** 3.21
CBGV	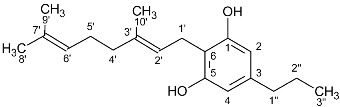	H-1′ **H-2′**	3.41 **5.27**
Δ8-THCV	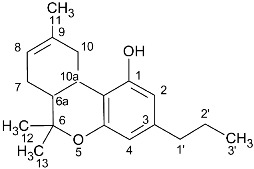	H-2 **H-8**	6.11 **5.45**

**Table 3:** COSY NMR peak correlation and calibration curves equations (mg/mL in tube).

Cannabinoids	Correlations (H-H)	Chemical Shift (δ)	Equation	R^2^
CBD	H-10 *trans*/H-9	4.66 ppm/1.66 ppm	C_CB_ = 0.0849 × I_CB_ + 0.0448	0.9993
CBDA	H-10 *trans*/H-10 *cis*	4.56 ppm/4.40 ppm	C_CB_ = 0.1696 × I_CB_ + 0.0163	0.9993
CBG & CBGA	H-2′/H-1′	5.31 ppm/3.42 ppm	C_CB_ = 0.0089 × I_CB_ − 0.0798	0.9995
CBN	H-10/H-8	8.18 ppm/7.07 ppm	C_CB_ = 0.19 × I_CB_ = 0.1554	0.9993
THCA & THC	H-10a/H-6a	3.25 ppm/1.68 ppm	C_CB_ = 0.0089 × I_CB_ + 0.0305	0.9998
Tyrosol (IS)	H-3, H-2/H-5, H-6	7.11 ppm/6.80 ppm		

**Table 4:** Comparison of ^1^H qNMR and ^1^H-^1^H-COSY qNMR methods.

	Sample	CBD (I)	CBD %	CBDA (I)	CBDA %	CBD&CBDA %	CBG&CBGA (I)	CBG&CBGA %
^1^H-NMR	R1N135	0.33	0.28	4.91	4.75	5.03	0.77	0.75
COSY NMR	R1N135	2.33	0.26	28.65	4.87	5.12	88.74	0.71
% Relative difference			7.69%		2.52%			5.63%
^1^H-NMR	R4N120	0.21	0.18	3.77	3.65	3.83	0.28	0.27
COSY NMR	R4N120	ND	ND	21.62	3.68	3.68	31.04	0.26
% Relative difference					0.82%	4.97%		3.84%
^1^H-NMR	R3N110	ND	ND	ND	ND	ND	3.28	3.19
COSY NMR	R3N110	ND	ND	ND	ND	ND	347.33	3.01
% Relative differene								5.98%

## Data Availability

The data presented in the method development and validations are available upon request from the corresponding author. The data on official samples from the applicability study are not publicly available due to government policy.

## Supplementary Materials

The following materials are available online at https://www.mdpi.com/article/10.3390/molecules27092965/s1: Table S1. COSY NMR chemical shift ranges, Table S2. Comparison of a carboxylated cannabis extract with different analytical methods, Tables S3 and S4. LOD, LOQ intra-day precision, inter-day precision and accuracy of ^1^H-NMR and ^1^H-^1^H COSY NMR quantitation methods, respectively, Table S5. Comparison of ^1^H qNMR and ^1^H-^1^H-COSY qNMR methods, Tables S6 and S7. ^1^H-NMR and COSY NMR acquisition parameters, respectively, Figures S1–S5. COSY NMR calibration curves, Figure S6. CBD, CBDA, CBN, CBDA, CBG, CBGA, and Δ9-THCA mixture COSY NMR spectra, Figures S7–S22. ^1^H-NMR spectra and structures of cannabinoids, Figure S23. HPLC–UV chromatograms of CBG, CBD, and CBN in different concentrations, and Figure S24. GC–MS chromatograms of CBD and CBG in different concentrations.
