# Supplementary material for: Direct Quantitation of Phytocannabinoids by One-Dimensional 1H qNMR and Two-Dimensional 1H-1H COSY qNMR in Complex Natural Mixtures
Source: Molecules. 2022 May 5;27(9):2965. doi: 10.3390/molecules27092965 (PMC9103933; doi:10.3390/molecules27092965)
Supplement: Supplementary file 1 [file molecules-27-02965-s001.zip › molecules-1677247-SM-proof.pdf]

molecules

MDPI

Article

Supplementary material: Direct quantitation of phytocannabinoids by one dimensional ^{1}H qNMR and two dimensional ^{1}H-^{1}H COSY qNMR in complex natural mixtures

Evangelos Dadiotis[1], Vangelis Mitsis[2], Eleni Melliou[1] and Prokopios Magiatis[1]

1 Laboratory of Pharmacognosy and Natural Products Chemistry, Department of Pharmacy, National and Kapodistrian University of Athens, Panepistimiopolis Zografou, Athens 15771, Greece
2 Ekati Alchemy Lab SL, Carretera Barcelona 11, Moia 08180 Spain

List of figures

3 ______ *Figure S1: COSY-NMR calibarition curve for CBN*
4 ______ *Figure S2: COSY-NMR calibarition curve for CBD*
4 ______ *Figure S3: COSY-NMR calibarition curve for CBDA*
5 ______ *Figure S4: COSY-NMR calibarition curve for CBG & CBGA*
5 ______ *Figure S5: COSY-NMR calibarition curve for Δ9-THC & Δ9-THCA*
6 ______ *Figure S6: CBD, CBDA, CBN, CBDA, CBG, CBGA, Δ9-THCA mixture COSY-NMR spectrum*
7 ______ *Figure S7: ^{1}H-NMR spectrum and structure of CBD*
8 ______ *Figure S8: ^{1}H-NMR spectrum and structure of CBDA*
9 ______ *Figure S9: ^{1}H-NMR spectrum and structure of CBG*
10 ______ *Figure S10: ^{1}H-NMR spectrum and structure of CBGA*
11 ______ *Figure S11: ^{1}H-NMR spectrum and structure of CBC*
12 ______ *Figure S12: ^{1}H-NMR spectrum and structure of CBCA*
13 ______ *Figure S13: ^{1}H-NMR spectrum and structure of Δ9-THC*
14 ______ *Figure S14: ^{1}H-NMR spectrum and structure of Δ9-THCA*
15 ______ *Figure S15: ^{1}H-NMR spectrum and structure of CBN*
16 ______ *Figure S16: ^{1}H-NMR spectrum and structure of Δ8-THC*
17 ______ *Figure S17: ^{1}H-NMR spectrum and structure of CBE*
18 ______ *Figure S18: ^{1}H-NMR spectrum and structure of CBGV*
19 ______ *Figure S19: ^{1}H-NMR spectrum and structure of Δ8-THCV*
20 ______ *Figure S20: ^{1}H-NMR spectrum and structure of CBDV*
21 ______ *Figure S21: ^{1}H-NMR spectrum and structure of CBDVA*
22 ______ *Figure S22: ^{1}H-NMR spectrum and structure of Δ9-THCVA*
23 ______ *Figure S23: HPLC- UV chromatograms of CBG, CBD and CBN in different concentrations*
24 ______ *Figure S24: GC-MS chromatograms of CBD and CBG in different concentrations*

List of tables

3 .... Table S1: COSY-NMR chemical shift ranges

25 .... Table S2: Comparison of a carboxylated cannabis extract with different analytical methods

25 .... Table S3: LOD, LOQ, intra-day precision, inter-day precision and accuracy of ^{1}H qNMR quantitation method

25 .... Table 4: LOD, LOQ intra-day precision, inter-day precision and accuracy of ^{1}H-^{1}H COSY NMR quantitation method

26 .... Table S5: Comparison of ^{1}H-qNMR and ^{1}H-^{1}H-COSY qNMR methods

27 .... Table S6: ^{1}H-NMR acquisition parameters

28 .... Table S7: COSY-NMR acquisition parameters

1. COSY-NMR chemical shift ranges, calibration curves and cannabinoids mixture COSY-NMR spectrum

1.1 COSY-NMR chemical shift ranges

Table S1: COSY-NMR chemical shift ranges used for integration

Cannabinoids	Correlations (H-H)	Chemical shift (δ) F1/ F2	Chemical shift range (δ) F1/ F2
**CBD**	H-10 *trans* / H-9	4.66 ppm / 1.66 ppm	1.735 - 1.577ppm / 4.711-4.649 ppm
**CBDA**	H-10 *trans* / H-10 *cis*	4.56 ppm / 4.40 ppm	4.607 - 4.488 ppm/ 4.448 - 4.488 ppm
**CBG & CBGA**	H-2′ / H-1′	3.42 ppm / 5.31 ppm	3.513-3.328 ppm / 5.351-5.251 ppm
**CBN**	H-10 / H-8	8.18 ppm / 7.07 ppm	8.247- 8.139 ppm/ 7.120 - 7.056 ppm
**THCA & THC**	H-10a / H – 6a	1.68 ppm/ 3.25 ppm	1.780 - 1.597 ppm/ 3.297 - 3.208 ppm
**Tyrosol (IS)**	H-3, H-2 / H-5, H-6	7.11 ppm / 6.80 ppm	7.181 - 7.036 ppm/ 6.843 - 6.767 ppm

1.2. COSY NMR calibration curves

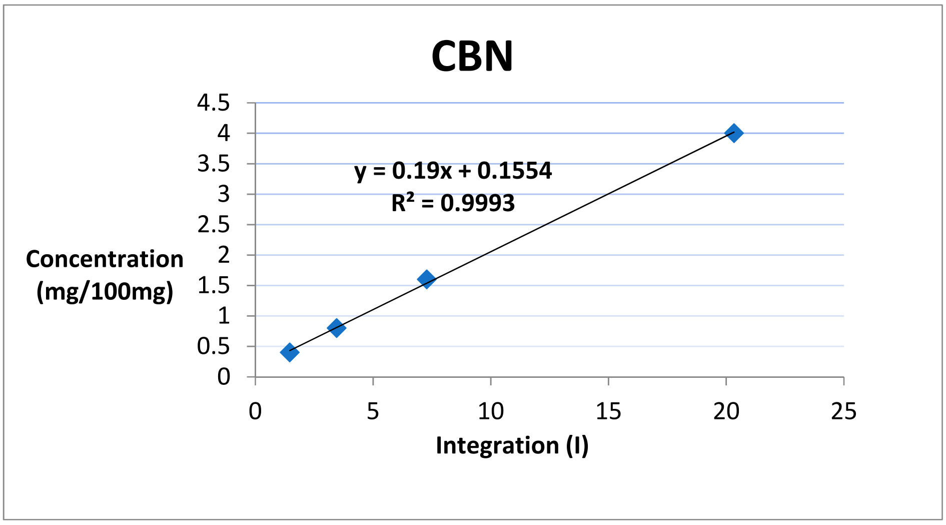

Figure S1: COSY-NMR calibarition curve for CBN

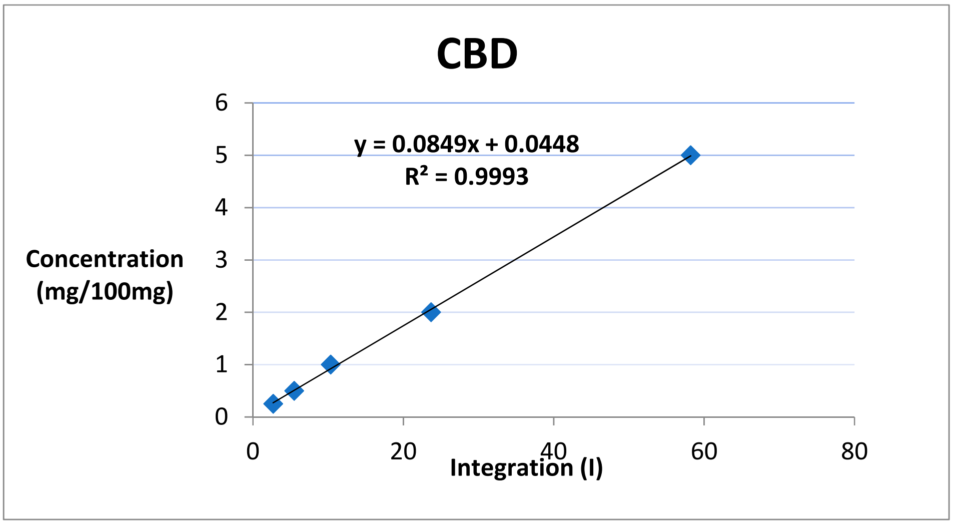

Figure S2: COSY-NMR calibarition curve for CBD

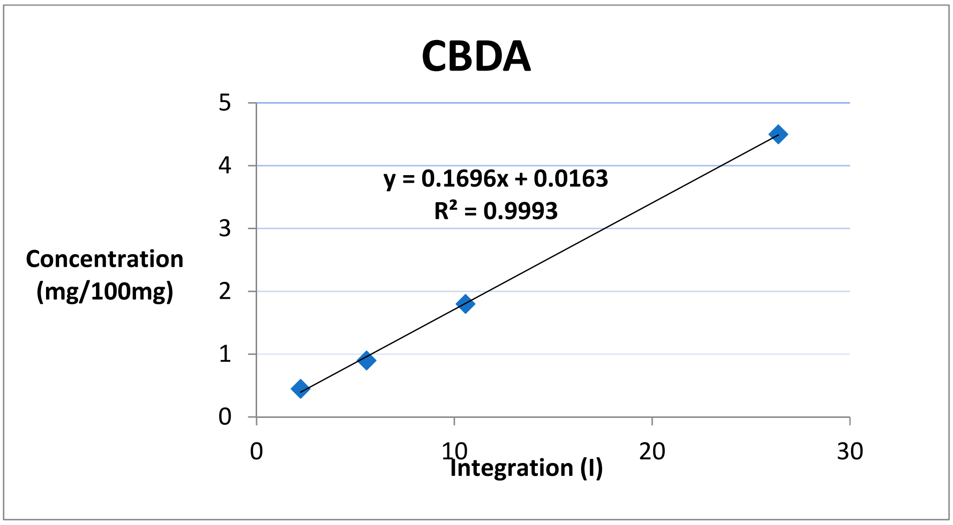

Figure S3: COSY-NMR calibarition curve for CBDA

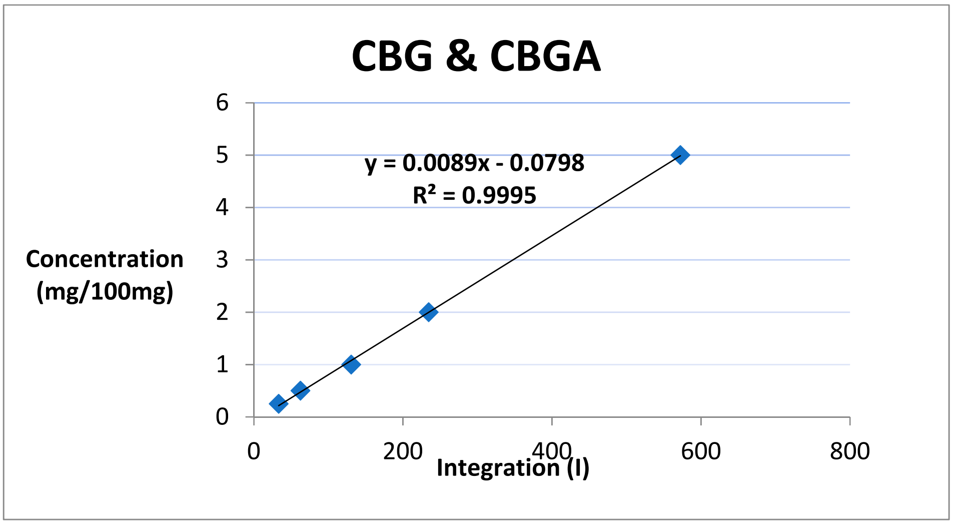

Figure S4: COSY-NMR calibarition curve for CBG & CBGA

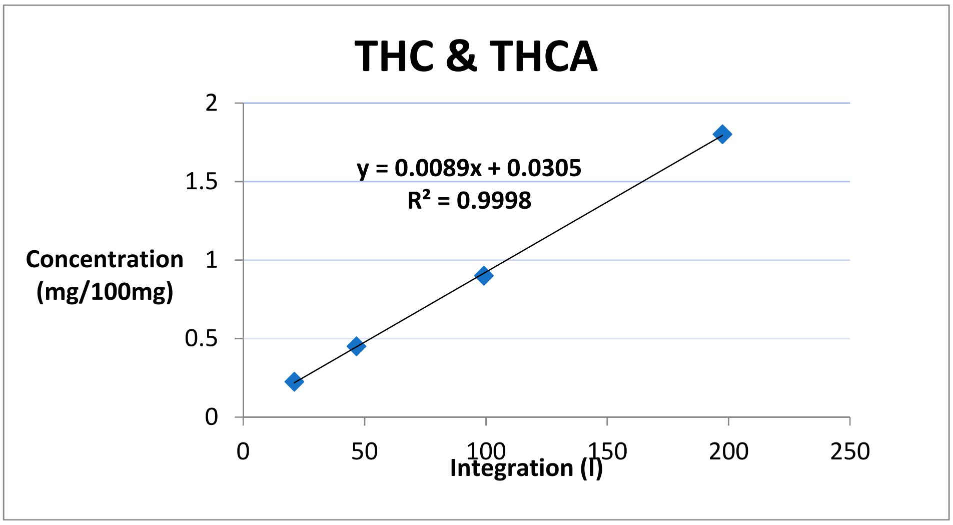

Figure S5: COSY-NMR calibarition curve for Δ9-THC & Δ9-THCA

1.3. CBD, CBDA, CBN, CBDA, CBG, CBGA, d9-THCA COSY-NMR spectrum

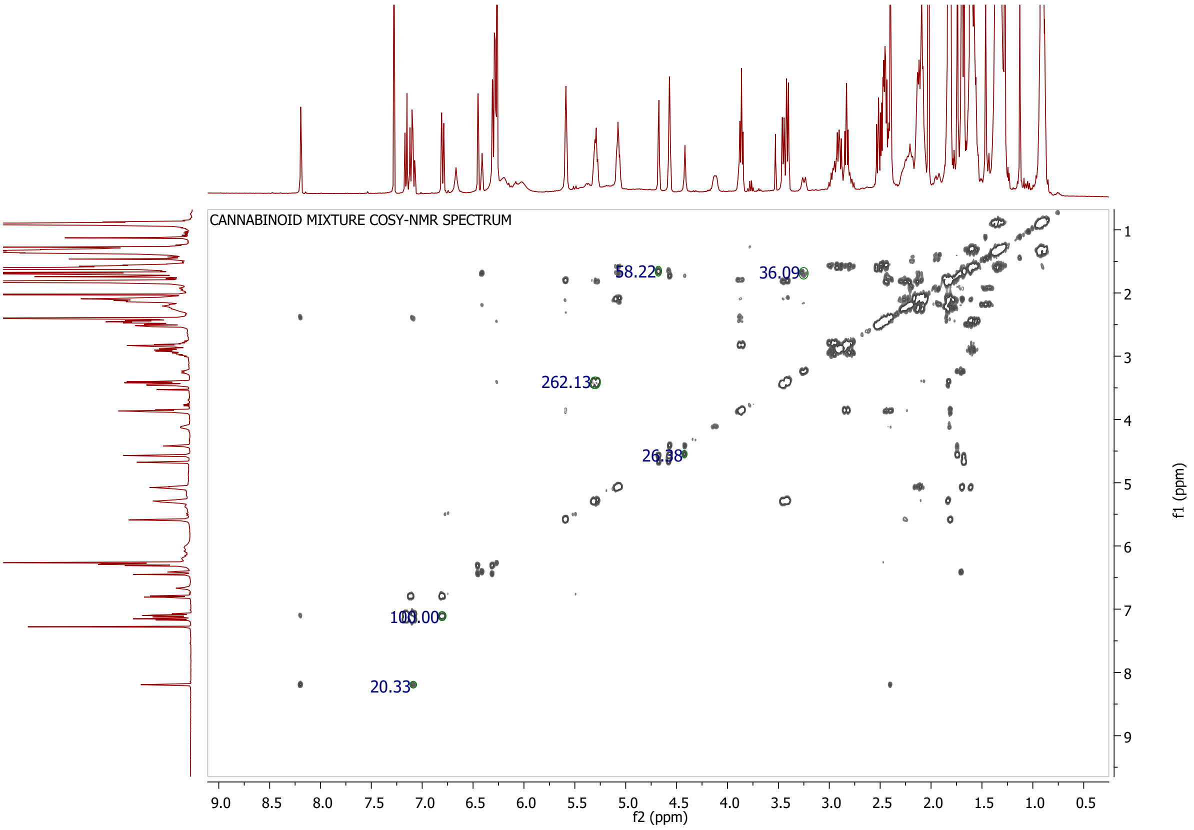

Figure S6: CBD, CBDA, CBN, CBDA, CBG, CBGA, Δ9-THCA mixture COSY-NMR spectrum

2. Cannabinoids ^{1}H-NMR spectrum, structure and chemical shifts

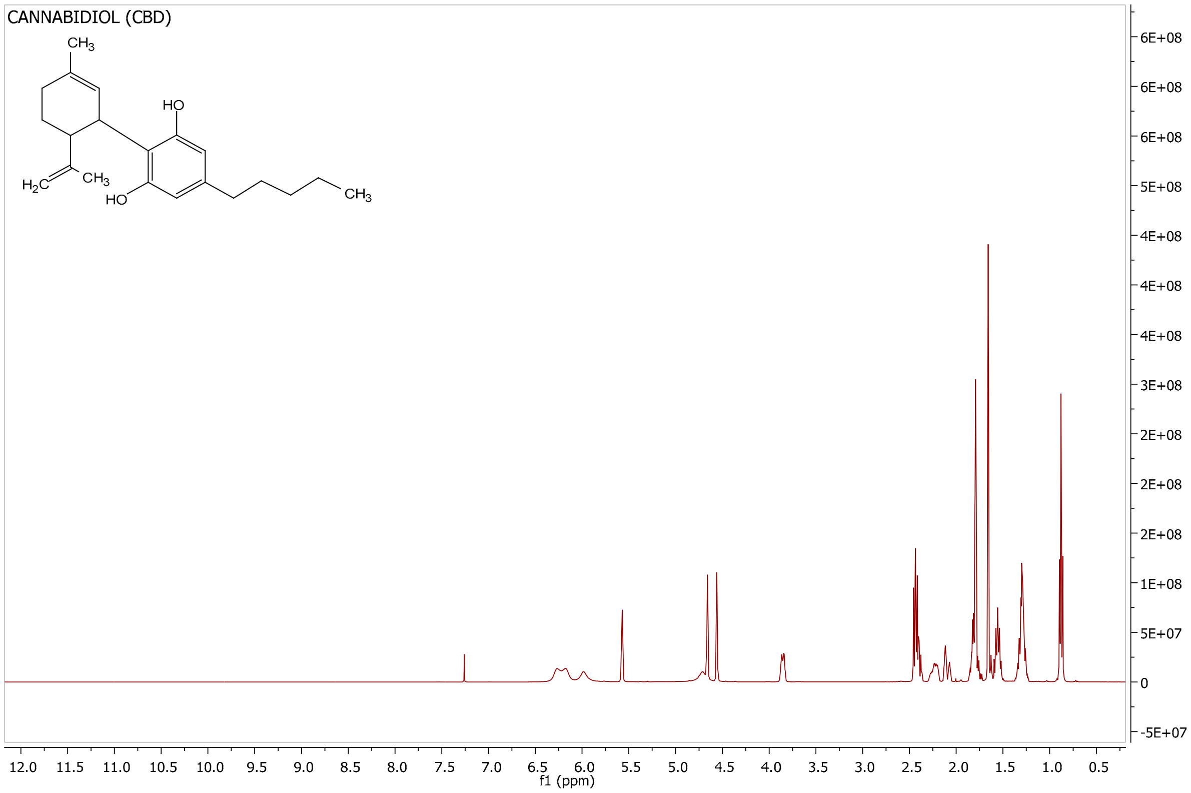

Figure S7: ^{1}H-NMR spectrum and structure of CBD

CBD			
**2**	5.56 (1H, s)	**1′′**	2.45 (2H, t, 7.5 Hz)
**3**	3.87 (1H, dm, 11.8 Hz)	**2′′**	1.55 (2H, q, 7.6 Hz)
**4**	2.40 (m)	**3′′**	1.30 (m)
**5**	1.84 (m)	**4′′**	1.30 (m)
**6**	2.22 (1H, m), 2.09 (1H, m)	**5′′**	0.88 (3H, t, 6.9 Hz)
**7**	1.80 (3H, s)	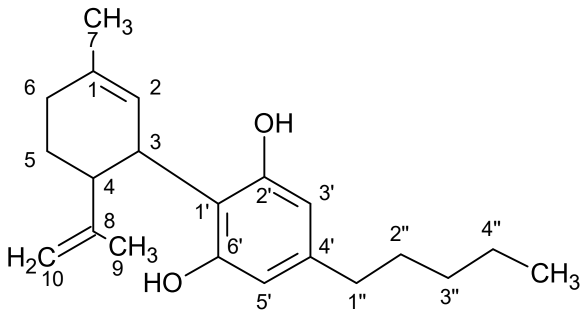	
**9**	1.66 (3H, s)		
**10**	4.66 (trans, 1H, m), 4.56 (cis, 1H, m)		
**3′**	6.29 (1H, brs)		
**4′**	6.20 (1H, brs)		

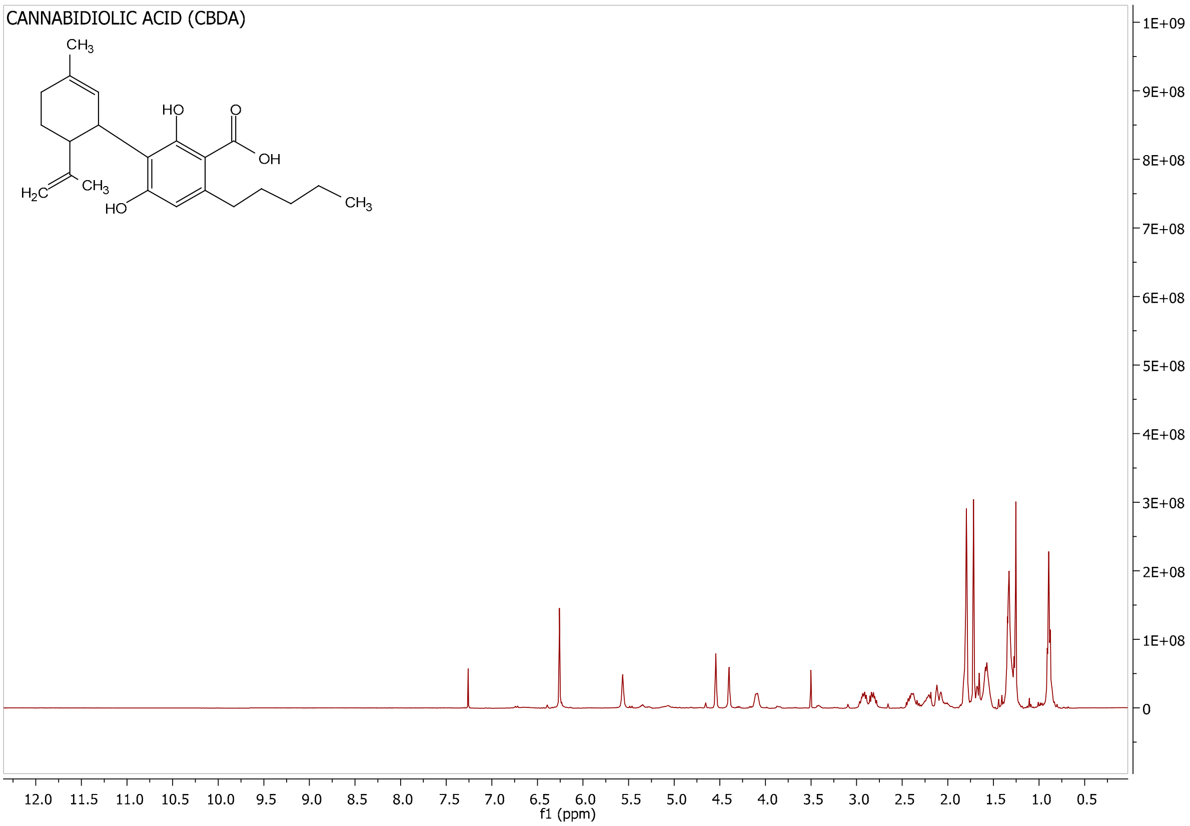

Figure S8: ¹H-NMR spectrum and structure of CBDA

**CBDA**			
**2**	5.56 (1H, s)	**1′′**	2.93 (1H, m), 2.83 (1H, m)
**3**	4.09 (1H, m)	**2′′**	1.57 (2H, m)
**4**	2.40 (m)	**3′′**	1.35 (4H, m)
**5**	1.86 (m)	**4′′**	1.35 (4H, m)
**6**	2.21 (1H, m), 2.10 (1H, m)	**5′′**	0.89 (3H, t, 6.9 Hz)
**7**	1.81 (3H, s)	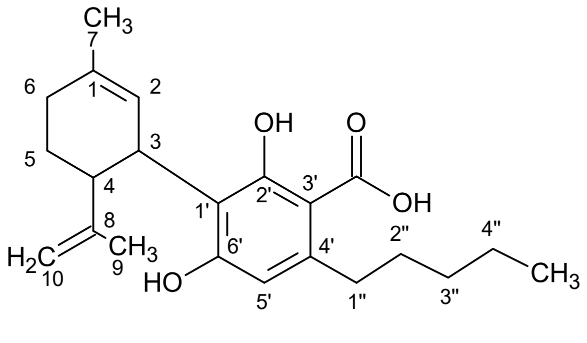	
**9**	1.72 (3H, s)		
**10**	4.56 (trans, 1H, m), 4.40 (cis, 1H, m)		
**3′**	-		
**4′**	6.26 (1H, s)		

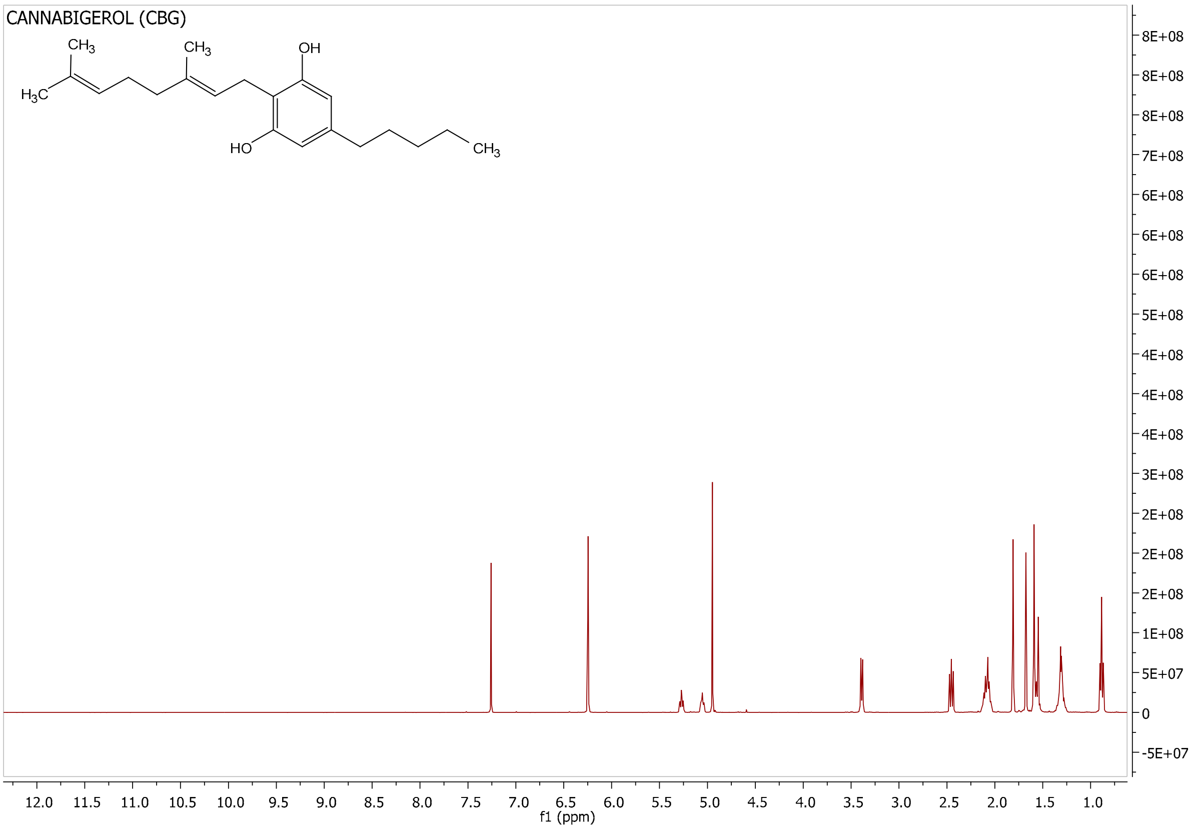

Figure S9: ^{1}H-NMR spectrum and structure of CBG

**CBG**			
**2**	6.26 (2H, s)	**1′′**	2.46 (2H, t, 7.5 Hz)
**4**	6.26 (2H, s)	**2′′**	1.56 (2H, m)
**1′**	3.41 (2H, d, 7.0 Hz)	**3′′**	1.31 (2H, m)
**2′**	5.29 (1H, m)	**4′′**	1.31 (2H, m)
**4′**	2.08 (2H, m)	**5′′**	0.88 (3H, t, 6.9 Hz)
**5′**	2.08 (2H, m)	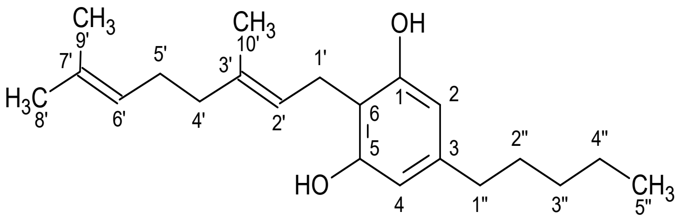	
**6′**	5.07 (1H, m)		
**8′**	1.61 (3H, s)		
**9′**	1.69 (3H, s)		
**10′**	1.83 (3H, s)		

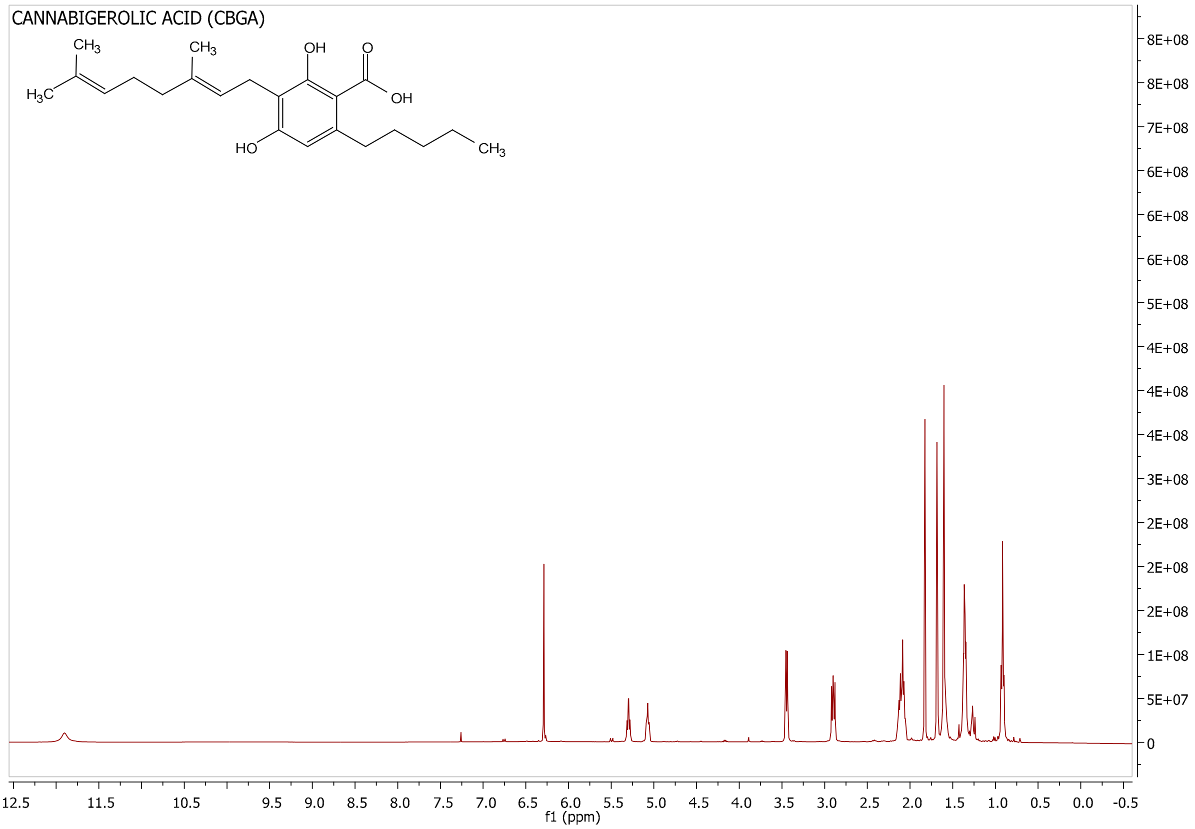

Figure S10: [1]**H-NMR spectrum and structure of CBGA**

CBGA			
**2**	-	**1′′**	2.88 (2H, t, 7.6 Hz)
**4**	6.29 (1H, s)	**2′′**	2.10 (2H, m)
**1′**	3.45 (2H, d, 7.0Hz)	**3′′**	1.35 (2H, m)
**2′**	5.29 (1H, t, 7.0 Hz)	**4′′**	1.35 (2H, m)
**4′**	2.09 (2H, t, 6.6 Hz)	**5′′**	0.90 (3H, t, 6.9 Hz)
**5′**	2.09 (2H, q, 6.5 Hz)	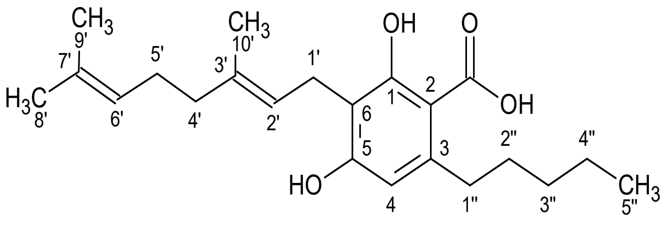	
**6′**	5.07 (1H, t, 6.6 Hz)		
**8′**	1.61 (3H, s)		
**9′**	1.69 (3H, s)		
**10′**	1.83 (3H, s)		

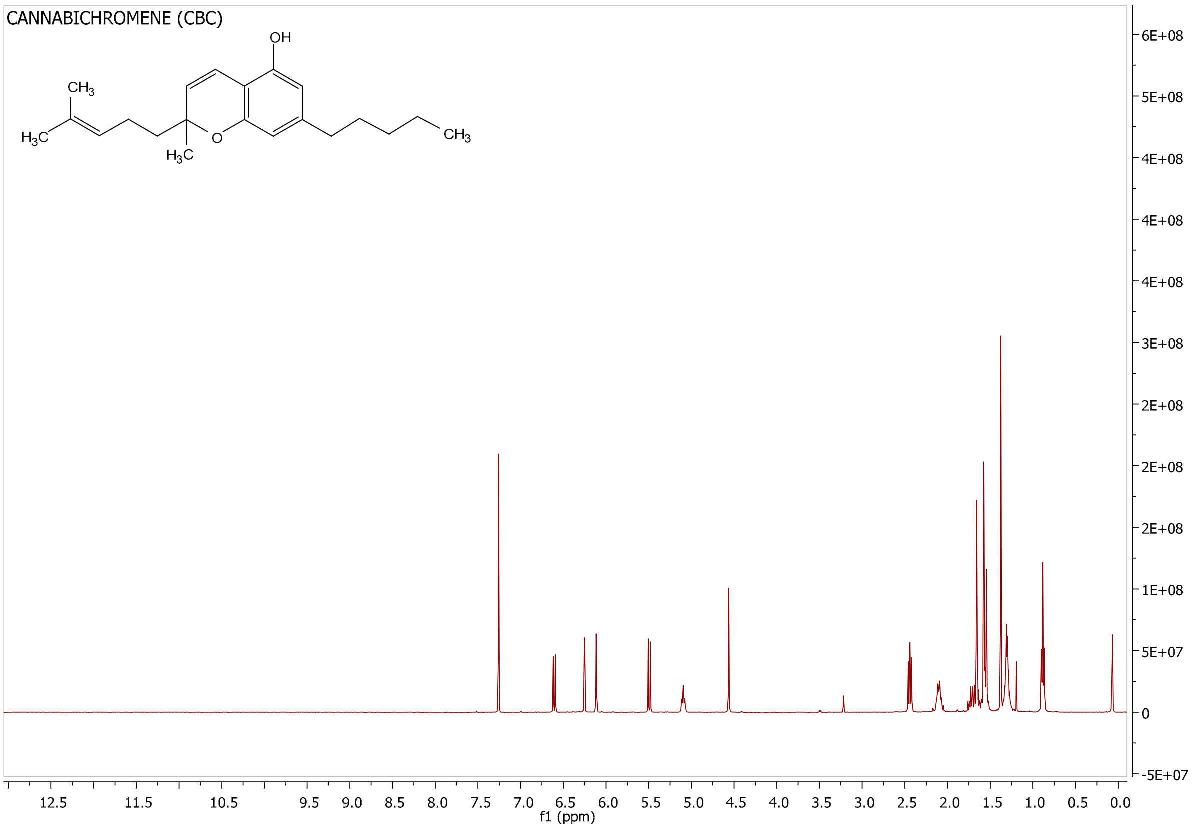

Figure S11: ^{1}H-NMR spectrum and structure of CBC

CBC			
**2**	6.11 (1H, s)	**1′′**	2.45 (2H, t, 7.5 Hz)
**4**	6.25 (1H, s)	**2′′**	1.55 (2H, q, 7.6 Hz)
**1′**	6.61 (1H, d, 10.0 Hz)	**3′′**	1.30 (m)
**2′**	5.49 (1H, d, 10.0 Hz)	**4′′**	1.30 (m)
**4′**	2.44 (2H, t, 6.9 Hz)	**5′′**	0.88 (3H, t, 6.9 Hz)
**5′**	2.12 (2H, m)	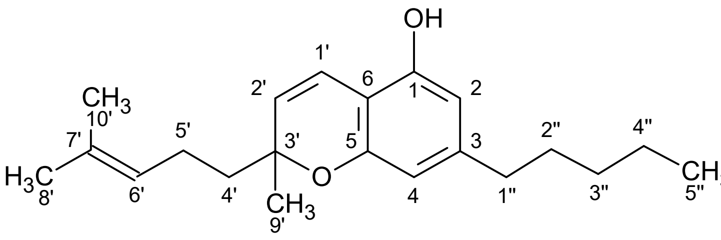	
**6′**	5.07 (1H, m)		
**8′**	1.58 (3H, s)		
**9′**	1.40 (3H, s)		
**10′**	1.66 (3H, s)		

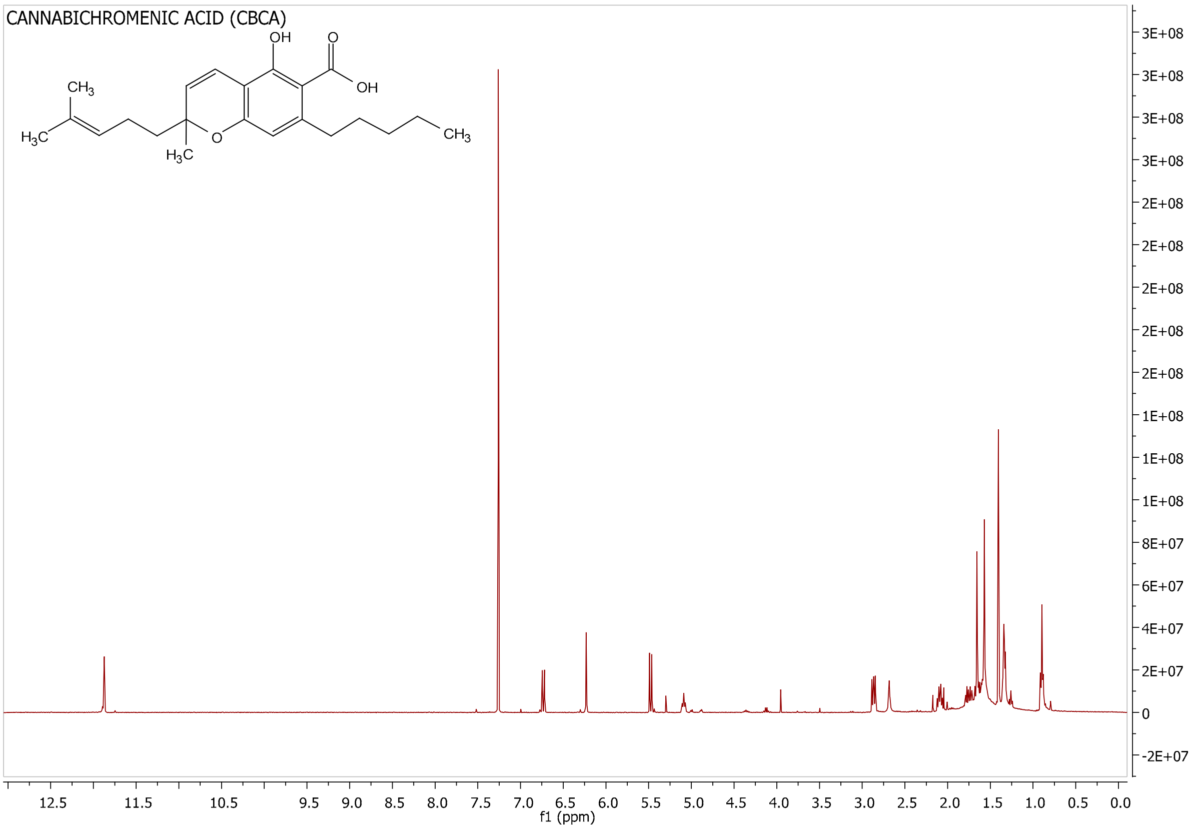

Figure S12: ^{1}H-NMR spectrum and structure of CBCA

CBCA			
**2**	-	**1″**	2.87 (2H, t, 7.5 Hz)
**4**	6.23 (1H, s)	**2″**	1.56 (2H, m)
**1′**	6.73 (1H, d, 10.0 Hz)	**3″**	1.34 (2H, m)
**2′**	5.48 (1H, d, 10.0 Hz)	**4″**	1.34 (2H, m)
**4′**	2.09 (2H, m)	**5″**	0.90 (3H, t, 6.9 Hz)
**5′**	2.09 (2H, m)		
**6′**	5.07 (1H, m)		
**8′**	1.57 (3H, s)		
**9′**	1.41 (3H, s)		
**10′**	1.66 (3H, s)		

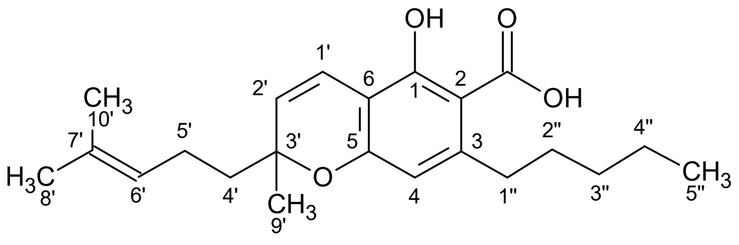

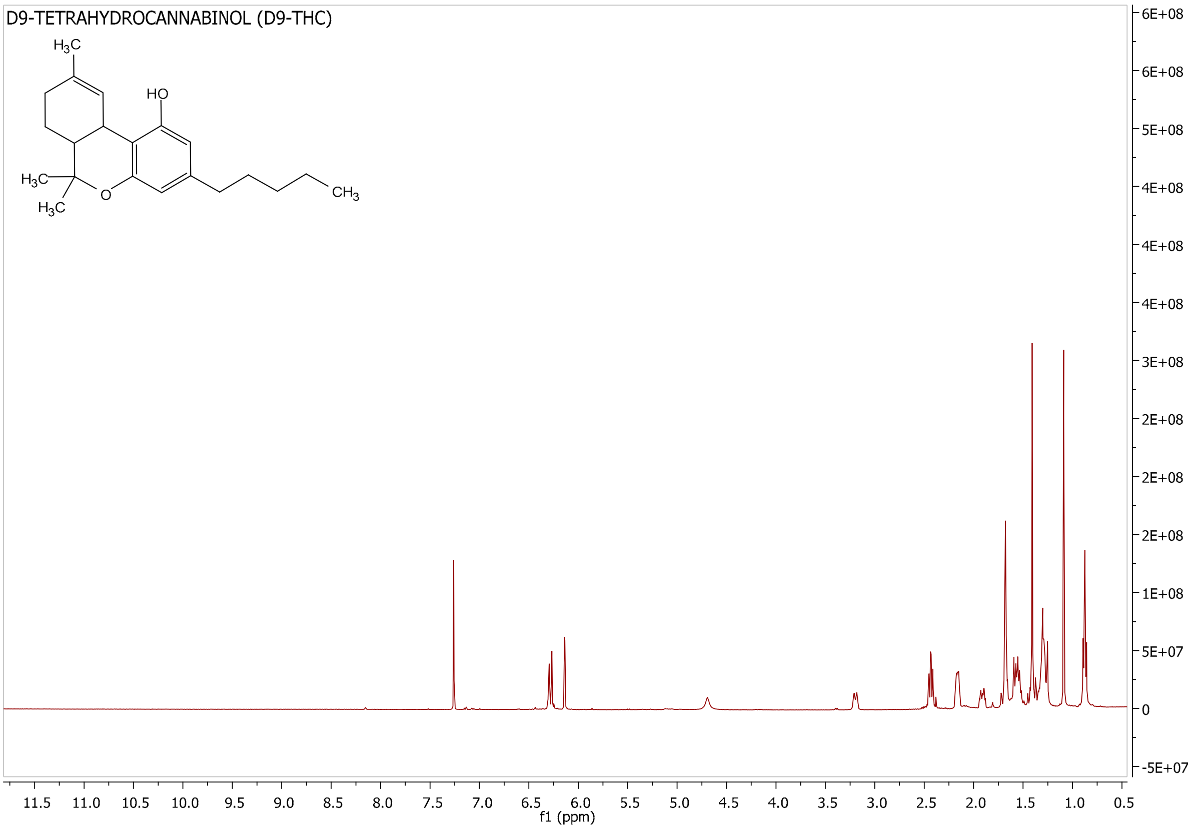

Figure S13: ^{1}H-NMR spectrum and structure of Δ9-THC

**Δ9-THC**			
**2**	6.14 (1H, d, 1.6 Hz	**1′**	2.45 (2H, td, 7.3 Hz, 1.6 Hz)
**4**	6.28 (1H, d, 1.6 Hz	**2′**	1.55 (2H, q, 7.8 Hz)
**6$^{\alpha}$**	1.69 (m)	**3′**	1.29 (2H, m)
**7**	1.90 (1H, m), 1.40 (m)	**4′**	1.29 (2H, m)
**8**	2.17 (2H, m)	**5′**	0.87 (3H, t, 7.0 Hz)
**10**	6.32 (1H, q, 1.6 Hz)	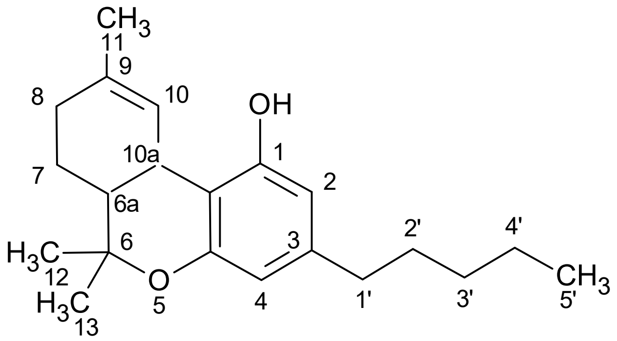	
**10$^{\alpha}$**	3.21 (1H, m)		
**11**	1.68 (3H, s)		
**12**	1.41 (3H, s)		
**13**	1.09 (3H, s		

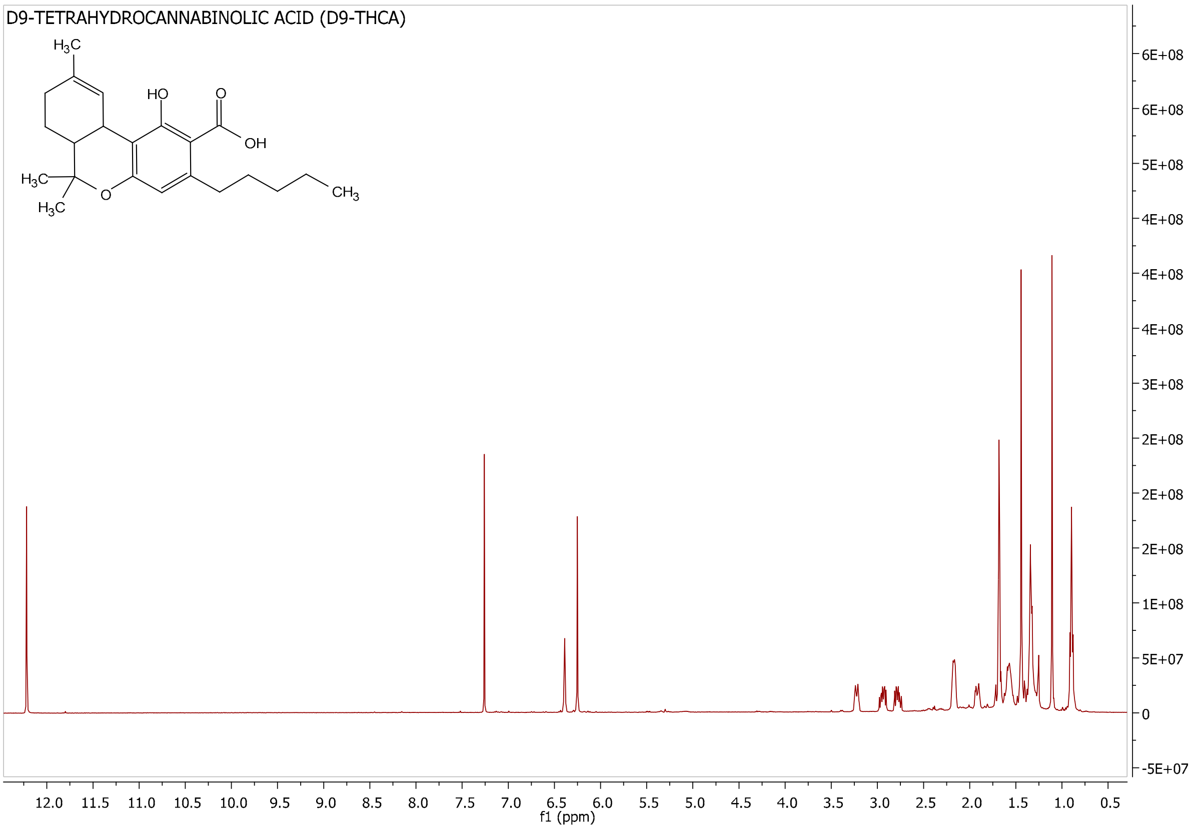

Figure S14: ^{1}H-NMR spectrum and structure of Δ9-THCA

Δ9-THCA			
**2**	-	**1′**	2.94 (1H, m), 2.78 (1H, m)
**4**	6.26 (1H, s	**2′**	1.57 (2H, m)
**6$^{\alpha}$**	1.67 (m)	**3′**	1.35 (2H, m)
**7**	1.92 (1H, m), 1.35 (m)	**4′**	1.35 (2H, m)
**8**	2.16 (2H, m)	**5′**	0.90 (3H, t, 6.9 Hz)
**10**	6.40 (1H, brs)	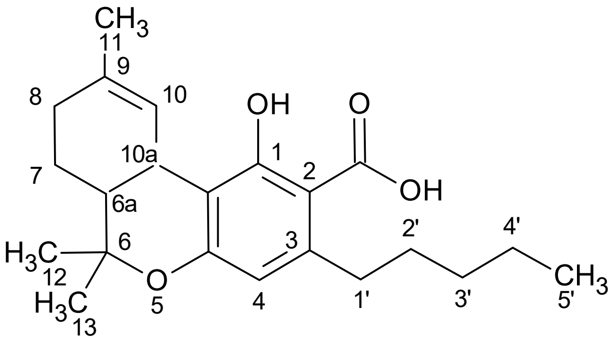	
**10$^{\alpha}$**	3.24 (1H, m)		
**11**	1.68 (3H, s)		
**12**	1.44 (3H, s)		
**13**	1.11 (3H, s)		

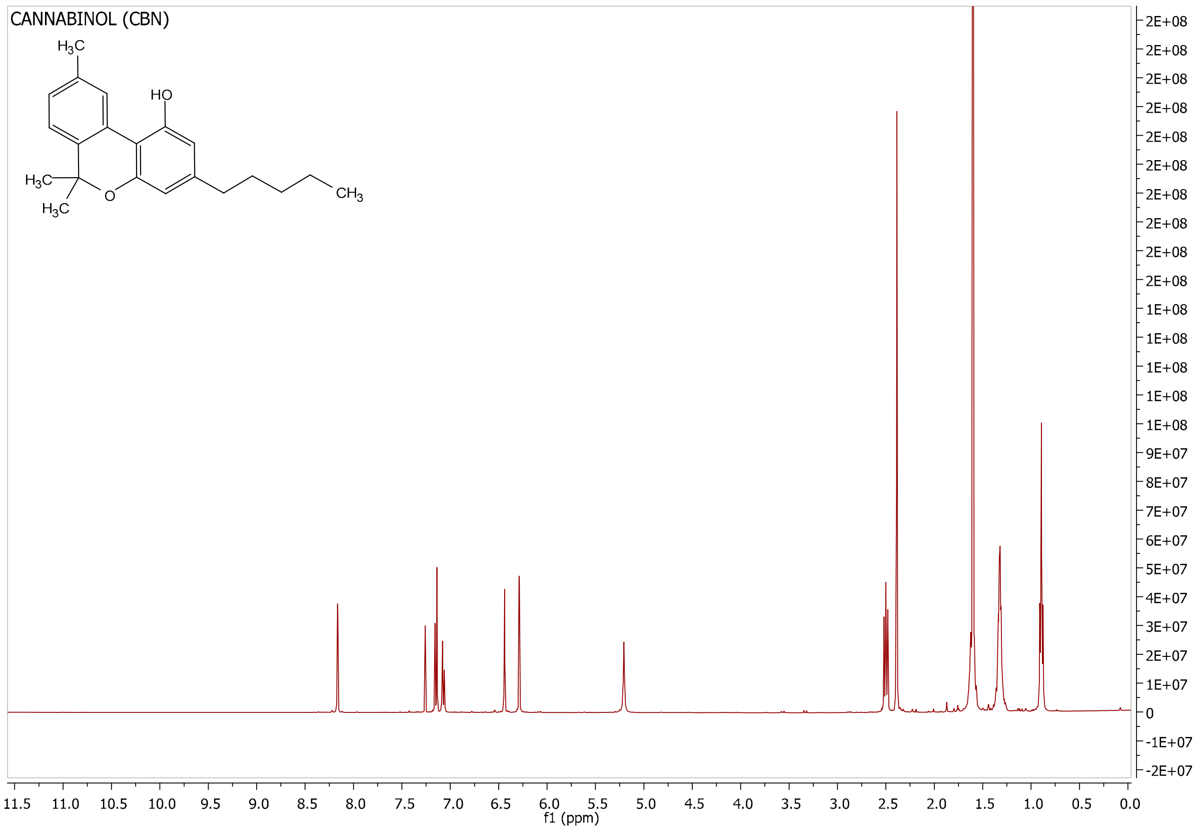

Figure S15: ^{1}H-NMR spectrum and structure of CBN

**CBN**			
**2**	6.29 (1H, d, 1.1 Hz)	**1′**	2.50 (2H, t, 7.5 Hz)
**4**	6.44 (1H, d, 1.1 Hz)	**2′**	1.63 (m)
**6$^{\alpha}$**	-	**3′**	1.32 (m)
**7**	7.14 (1H, d, 7.9 Hz)	**4′**	1.32 (m)
**8**	7.07 (1H, d, 7.9 Hz)	**5′**	0.89 (3H, t, 6.8 Hz)
**10**	8.18 (1H, s)	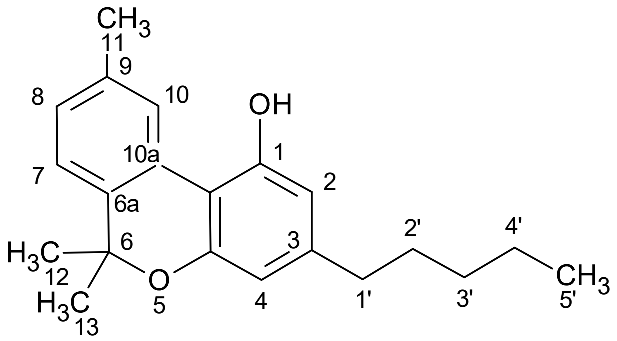	
**10$^{\alpha}$**	2.38 (3H, s)		
**11**	1.60 (6H, s)		
**12**	1.60 (6H, s)		
**13**	6.29 (1H, d, 1.1 Hz)		

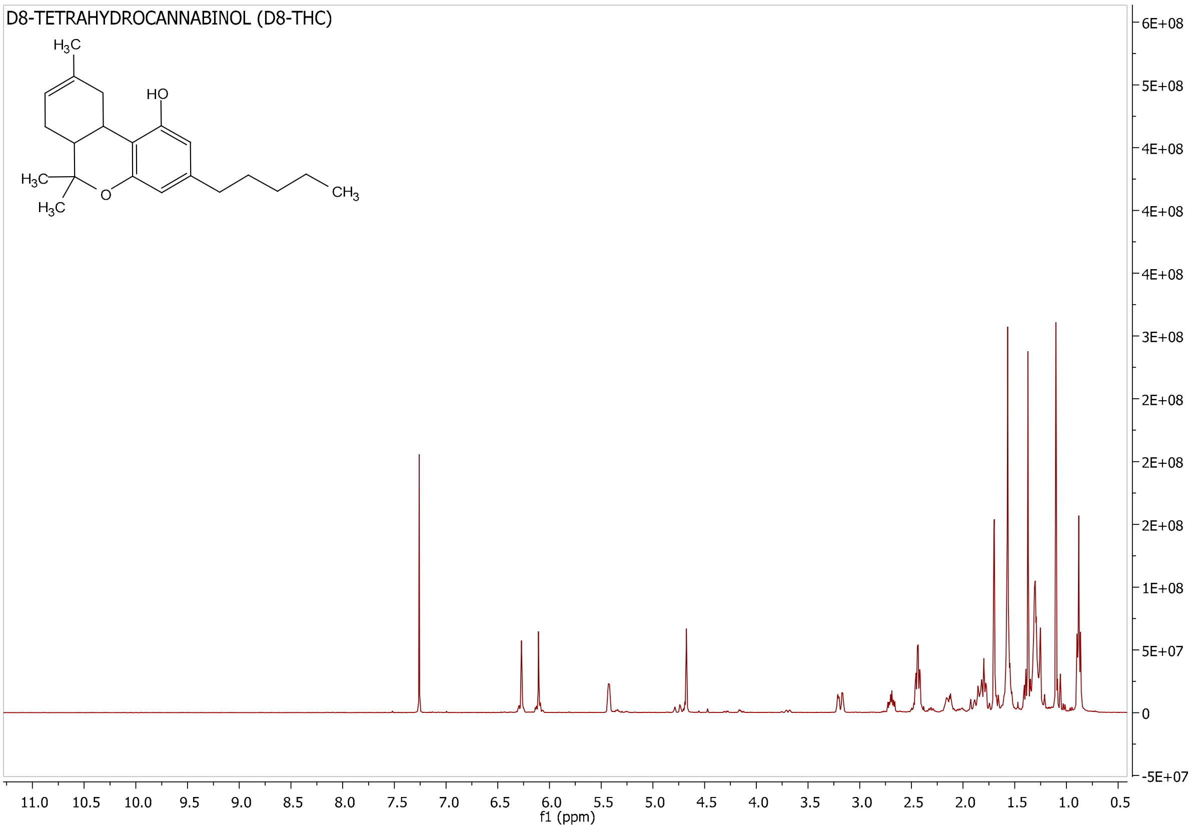

Figure S16: ¹H-NMR spectrum and structure of Δ8-THC

**Δ8-THC**			
**2**	6.11 (1H, d, 1.6 Hz)	**1′**	2.44 (2H, td, 8.3 Hz, 2.1 Hz)
**4**	6.27 (1H, d, 1.5 Hz)	**2′**	1.56 (2H, q, 7.6 Hz)
**6ᵅ**	1.80 (m)	**3′**	1.32 (m)
**7**	2.13 (1H, m), 1.64 (1H, s)	**4′**	1.32 (m)
**8**	5.43 (1H, brd, 4.8 Hz)	**5′**	0.88 (3H, t, 7.1 Hz)
**10**	3.19 (2H, dd, 16.5 Hz, 3.7 Hz)	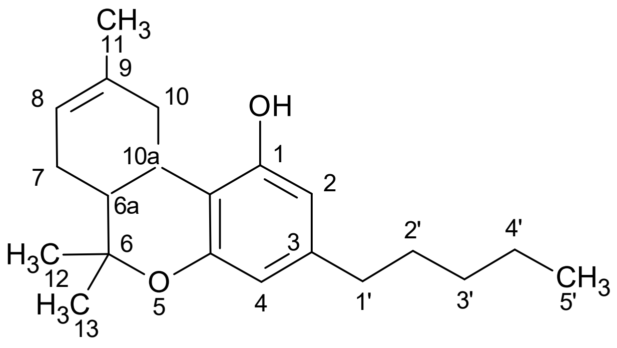	
**10ᵅ**	1.70 (3H, s)		
**11**	1.38 (3H, s)		
**12**	1.10 (3H, s)		
**13**	6.11 (1H, d, 1.6 Hz)		

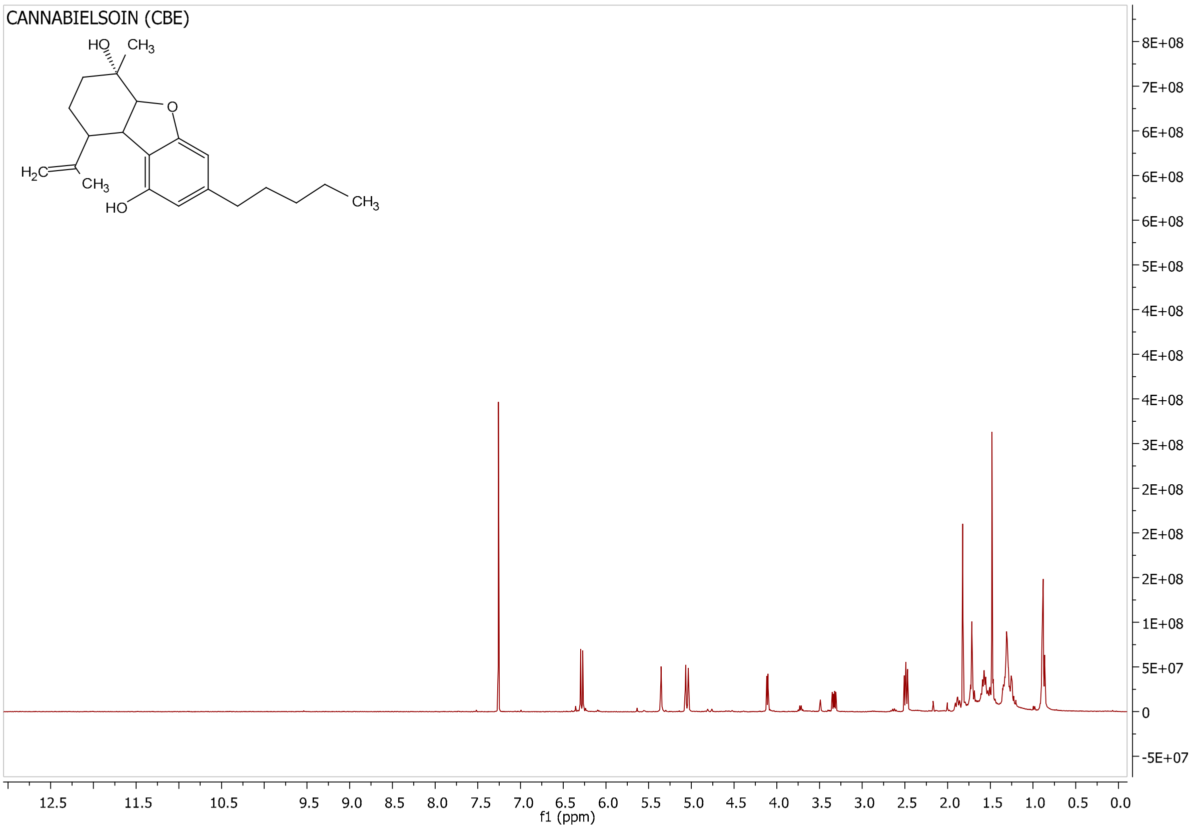

Figure S17: ^{1}H-NMR spectrum and structure of CBE

**CBE**			
**2**	4.11 (1H, d, 6.0 Hz)	**1′′**	2.49 (2H, t, 8.0 Hz)
**3**	3.33 (1H, dd, 11.0 Hz, 6.0 Hz)	**2′′**	1.58 (2H, m)
**4**	1.89 (1H, m)	**3′′**	1.31 (2H, m)
**5**	1.72 (2H, m)	**4′′**	1.31 (2H, m)
**6**	1.51 (2H, m)	**5′′**	0.88 (3H,t,7.0)
**7**	1.47 (3H, s)	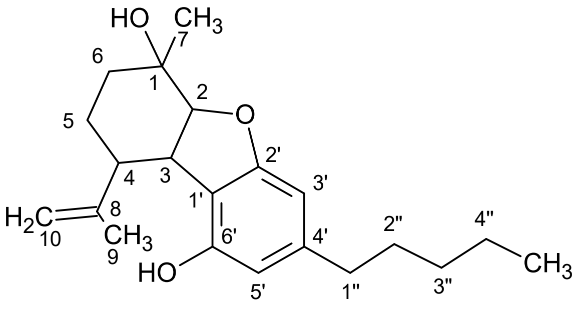	
**9**	1.84 (3H, s)		
**10**	5.04 (1H, m)<br>5.07 (1H, s)		
**3′**	6.30 (1H, s)		
**4′**	6.27 (1H, s)		

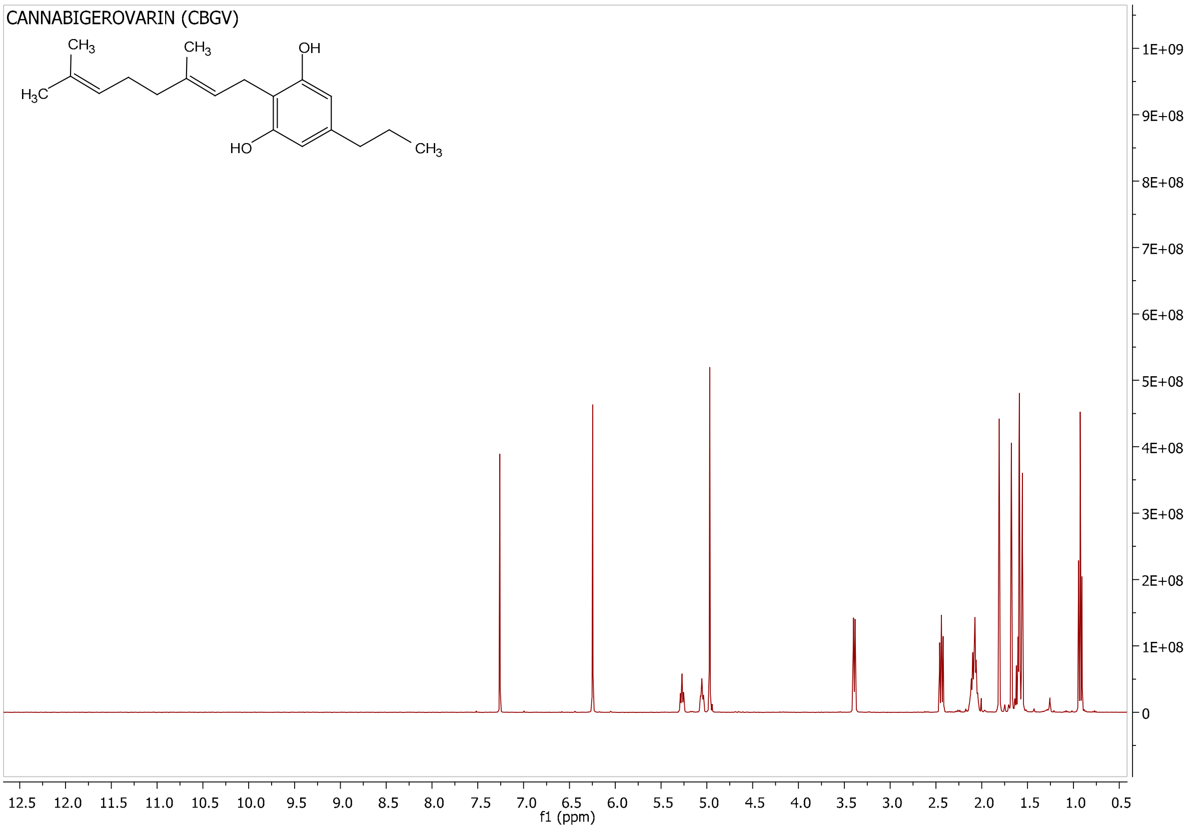

Figure S18: ¹H-NMR spectrum and structure of CBGV

**CBGV**			
**2**	6.26 (2H, s)	**1′′**	2.46 (2H, t, 7.5 Hz)
**4**	6.26 (2H, s)	**2′′**	1.56 (2H, m)
**1′**	3.41 (2H, d, 7.0Hz)	**3′′**	0.92 (3H, t, 6.9 Hz)
**2′**	5.29 (1H, m)	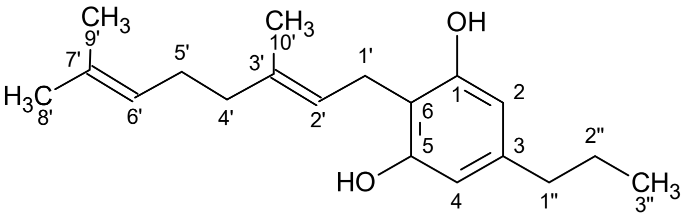	
**4′**	2.08 (2H, m)		
**5′**	2.08 (2H, m)		
**6′**	5.07 (1H, m)		
**8′**	1.61 (3H, s)		
**9′**	1.69 (3H, s)		
**10′**	1.83 (3H, s)		

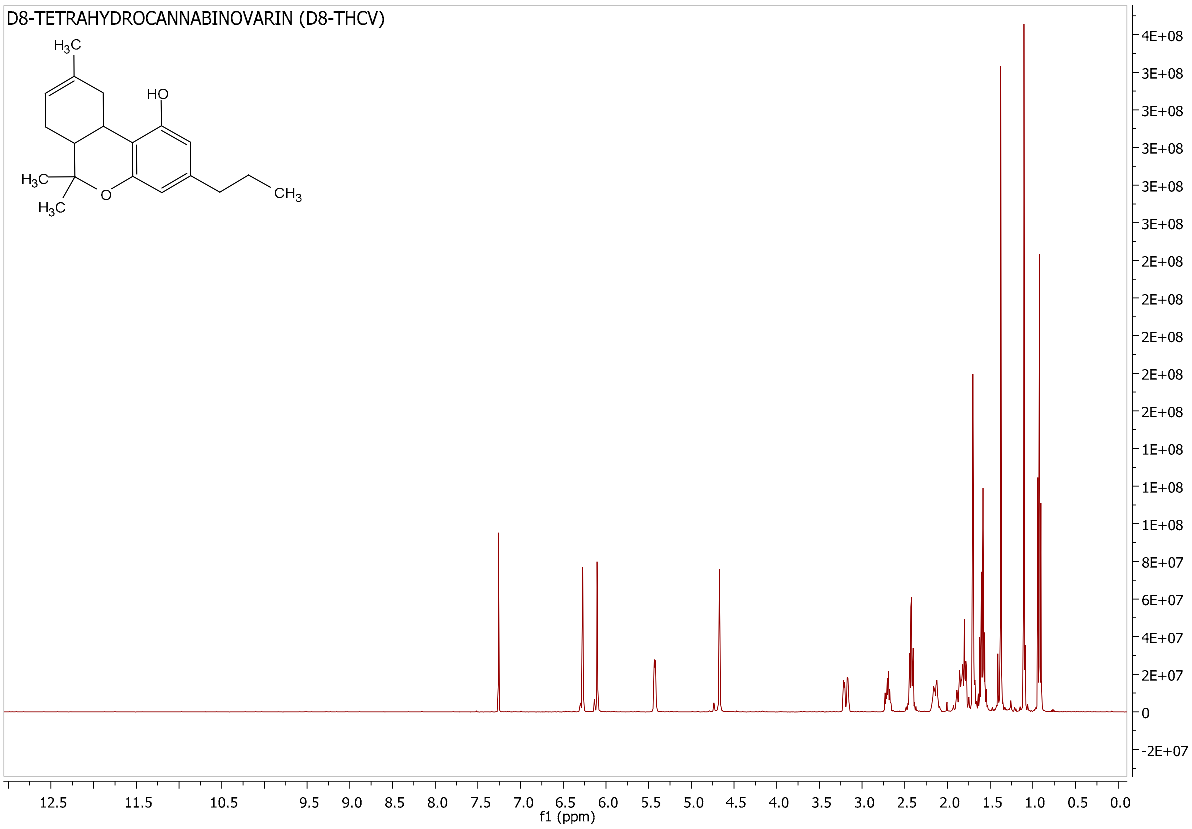

Figure S19: ^{1}H-NMR spectrum and structure of Δ8-THCV

**Δ8-THCV**			
**2**	6.11 (1H, d, 1.6 Hz)	**1′**	2.44 (2H, td, 8.3 Hz, 2.1 Hz)
**4**	6.27 (1H, d, 1.5 Hz)	**2′**	1.56 (2H, q, 7.6 Hz)
**6$^{\alpha}$**	1.80 (m)	**3′**	0.92 (3H, t, 7.1 Hz)
**7**	2.13 (1H, m), 1.64 (1H, s)	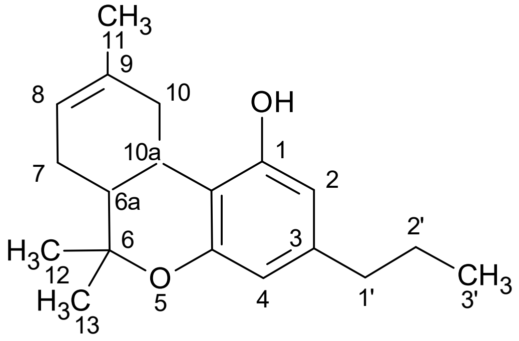	
**8**	5.43 (1H, brd, 4.8 Hz)		
**10**	3.19 (2H, dd, 16.5 Hz, 3.7 Hz)		
**10$^{\alpha}$**	1.70 (3H, s)		
**11**	1.38 (3H, s)		
**12**	1.10 (3H, s)		
**13**	6.11 (1H, d, 1.6 Hz)		

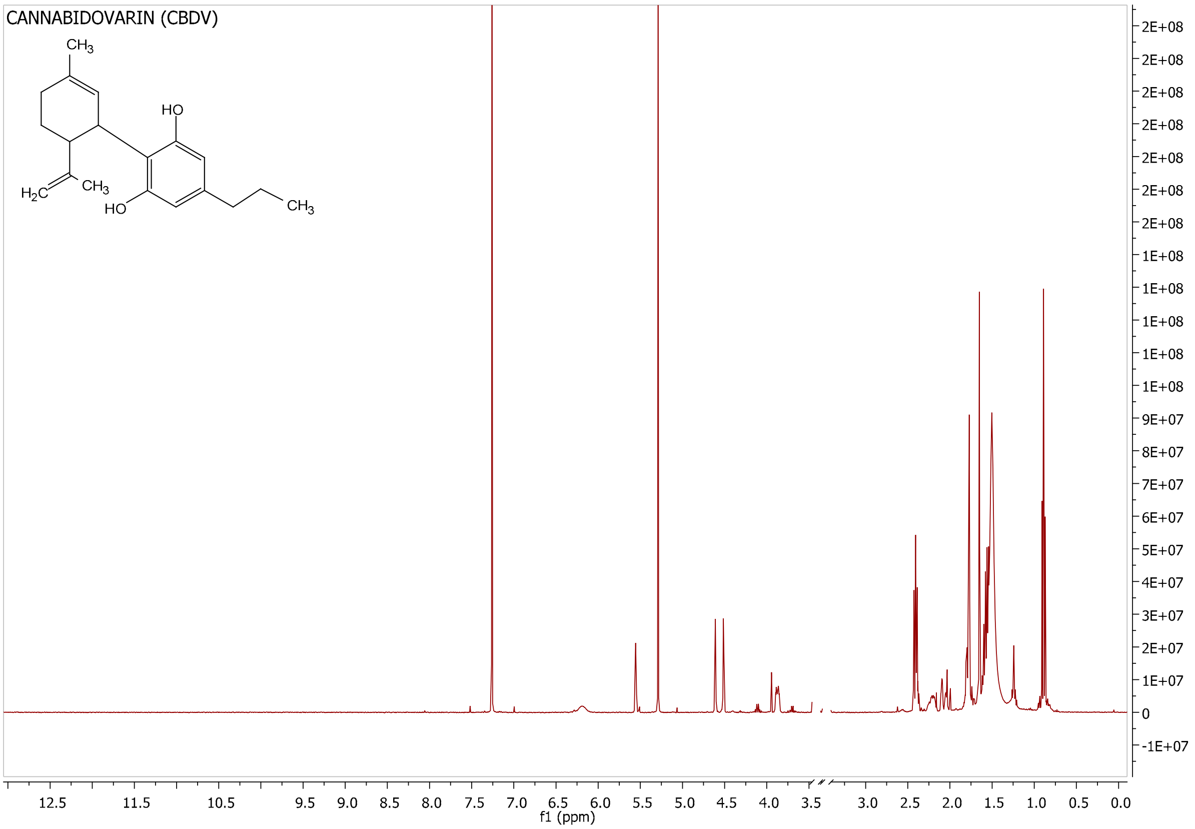

Figure S20: ¹H-NMR spectrum and structure of CBDV

**CBDV**			
**2**	5.57 (1H, s)	**1″**	2.45 (2H, t, 7.5 Hz)
**3**	3.87 (1H, dm, 11.8 Hz)	**2″**	1.55 (2H, q, 7.6 Hz)
**4**	2.40 (m)	**3″**	0.88 (3H, t, 6.9 Hz)
**5**	1.84 (m)	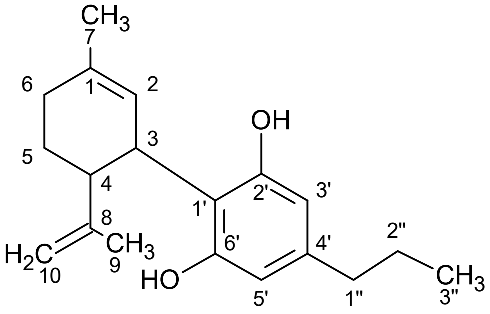	
**6**	2.22 (1H, m), 2.09 (1H, m)		
**7**	1.80 (3H, s)		
**9**	1.66 (3H, s)		
**10**	4.61 (trans, 1H, m), 4.52 (cis, 1H, m)		
**3′**	6.19 (1H, brs)		
**4′**	6.19 (1H, brs)		

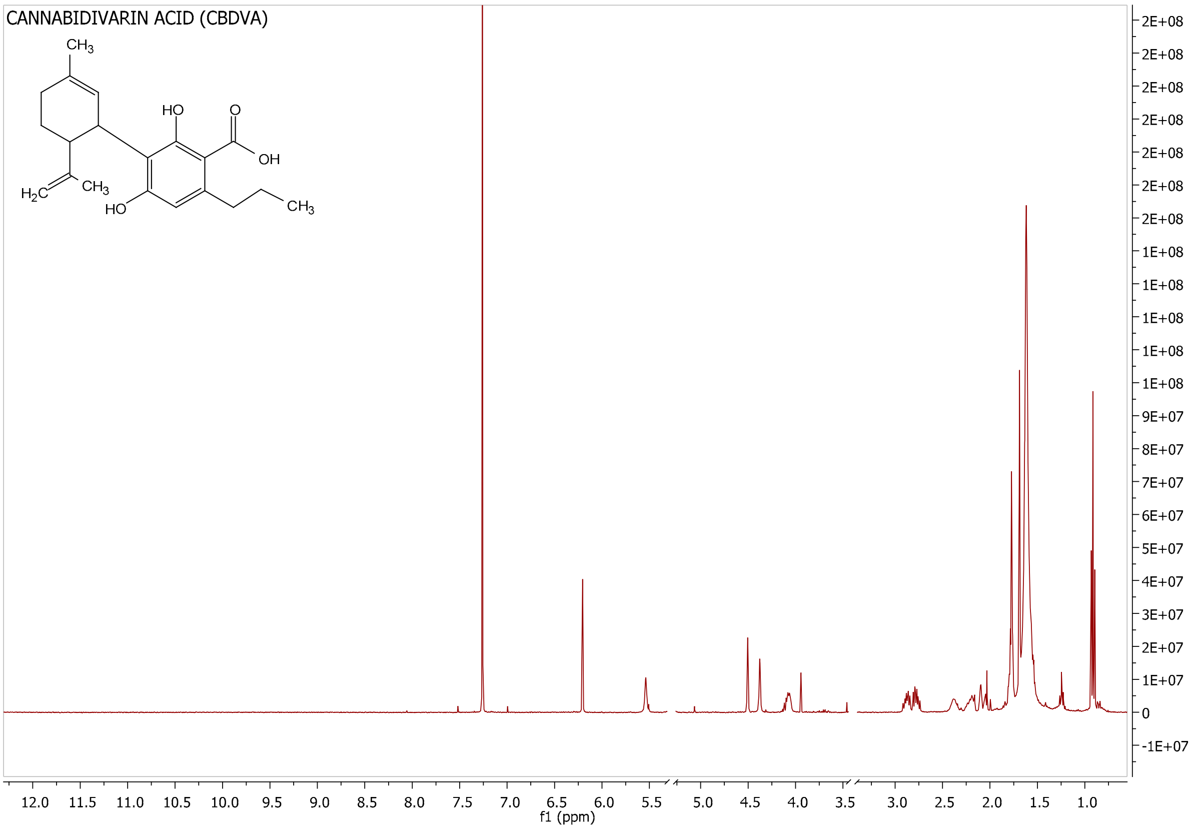

Figure S21: ^{1}H-NMR spectrum and structure of CBDVA

<table>
<tr><th colspan="4">CBDVA</th></tr>
<tr><td>2</td><td>5.54 (1H, s)</td><td>1′′</td><td>2.93 (1H, m), 2.83 (1H, m)</td></tr>
<tr><td>3</td><td>4.07 (1H, m)</td><td>2′′</td><td>1.57 (2H, m)</td></tr>
<tr><td>4</td><td>2.40 (m)</td><td>3′′</td><td>0.91 (3H, t, 6.9 Hz)</td></tr>
<tr><td>5</td><td>1.86 (m)</td><td colspan="2" rowspan="7">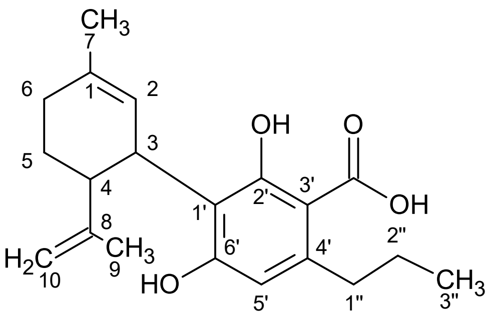</td></tr>
<tr><td>6</td><td>2.21 (1H, m), 2.10 (1H, m)</td></tr>
<tr><td>7</td><td>1.77 (3H, s)</td></tr>
<tr><td>9</td><td>1.69 (3H, s)</td></tr>
<tr><td>10</td><td>4.50 (trans, 1H, m), 4.38 (cis, 1H, m)</td></tr>
<tr><td>3′</td><td>-</td></tr>
<tr><td>4′</td><td>6.21 (1H, s)</td></tr>
</table>

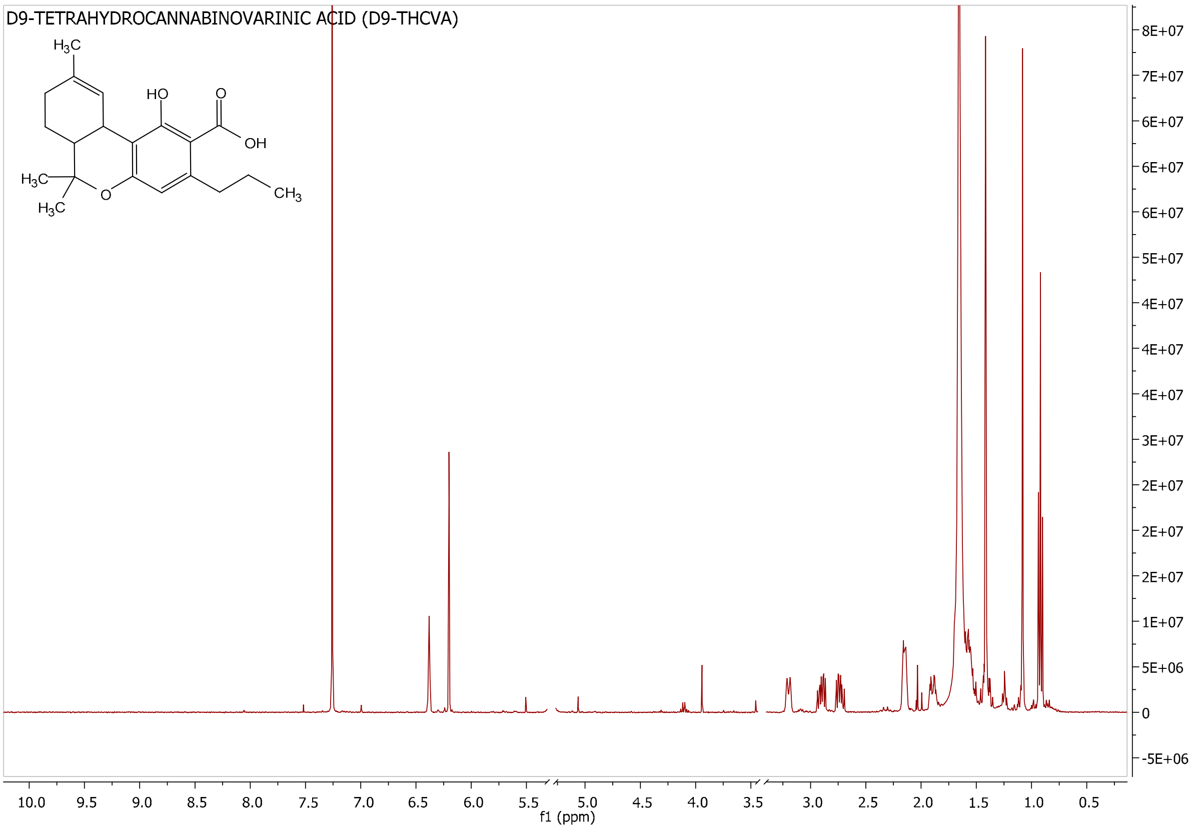

Figure S22: ^{1}H-NMR spectrum and structure of Δ9-THCVA

**Δ9-THCVA**			
**2**	-	**1′**	2.90 (1H, m), 2.74 (1H, m)
**4**	6.20 (1H, s	**2′**	1.57 (2H, m)
**6$^{\alpha}$**	1.67 (m)	**3′**	0.92 (3H, t, 6.9 Hz)
**7**	1.92 (1H, m), 1.35 (m)		
**8**	2.14 (2H, m)		
**10**	6.39 (1H, brs)		
**10$^{\alpha}$**	3.21 (1H, m)		
**11**	1.68 (3H, s)		
**12**	1.42 (3H, s)		
**13**	1.08 (3H, s)		

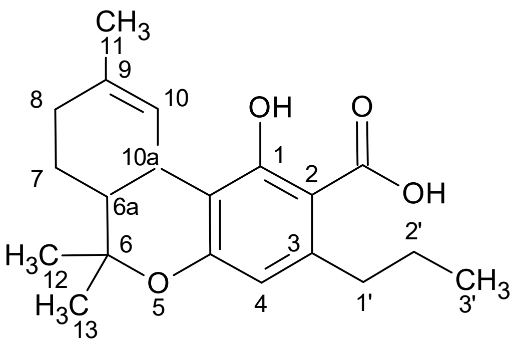

3. GC-MS and HPLC analysis

3.1. HPLC-UV ANALYSIS

HPLC quantification was performed with the Beckman Autosampler 507, 126 Solvent Module and 166 Detector (System Gold), with a Rainin C18 analytical column (5µm, 200mm × 5mm). 80:20 acetonitrile / water with 0.2% formic acid used as the mobile phase with isocratic elution. The wavelength was set at 228nm and the duration of the method for quantitation of CBD, CBG and CBN was 10 min. Concentrations of 10ppm, 25ppm, 50ppm, 75ppm, 100ppm and 150ppm of CBD, CBG and CBN solution in acetonitrile were prepared for the calibration curves. Then, 10µl of the solutions were injected.

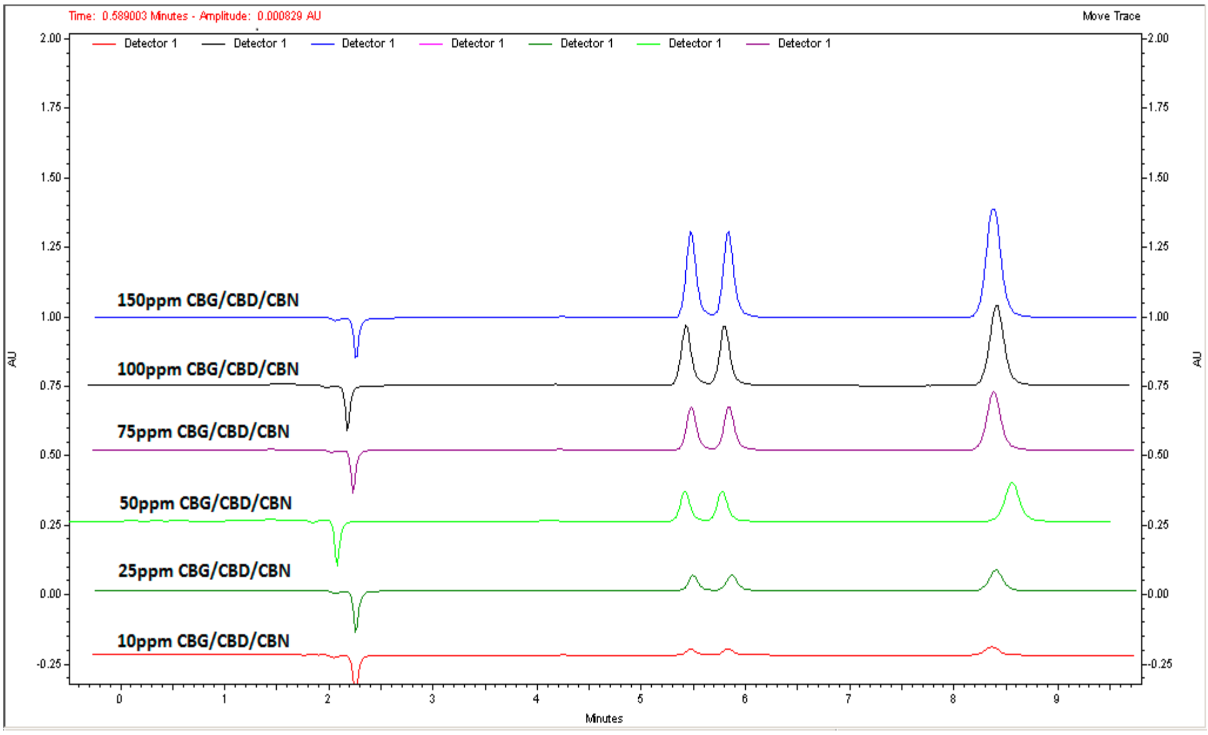

Figure S23: HPLC- UV chromatograms of CBG, CBD and CBN in different concentrations

3.2. GC-MS ANALYSIS

GC-MS quantification was performed with the Agilent Technologies 7820A GC System gas chromatograph in combination with the Agilent Technologies 5977B MSD mass spectrometer, with a HP 5MS 30m, 0.25 mm, 0.25m capillary column and helium gas carrier (1ml / min). The quantitation using GC-MS was performed using reference standards of cannabidiol (CBD) and cannabigerol (CBG), to generate calibration curves with the use of squalene as an internal standard, which is used as an internal standard in the analysis method. Greek legislation. Concentrations of 25 ppm, 50 ppm, 100 ppm, 200 ppm, 500 ppm and 750 ppm of CBD and CBG solution in acetonitrile were prepared for the reference curve, to which a constant amount of squalene solution was added to each solution and the final solutions were injected.

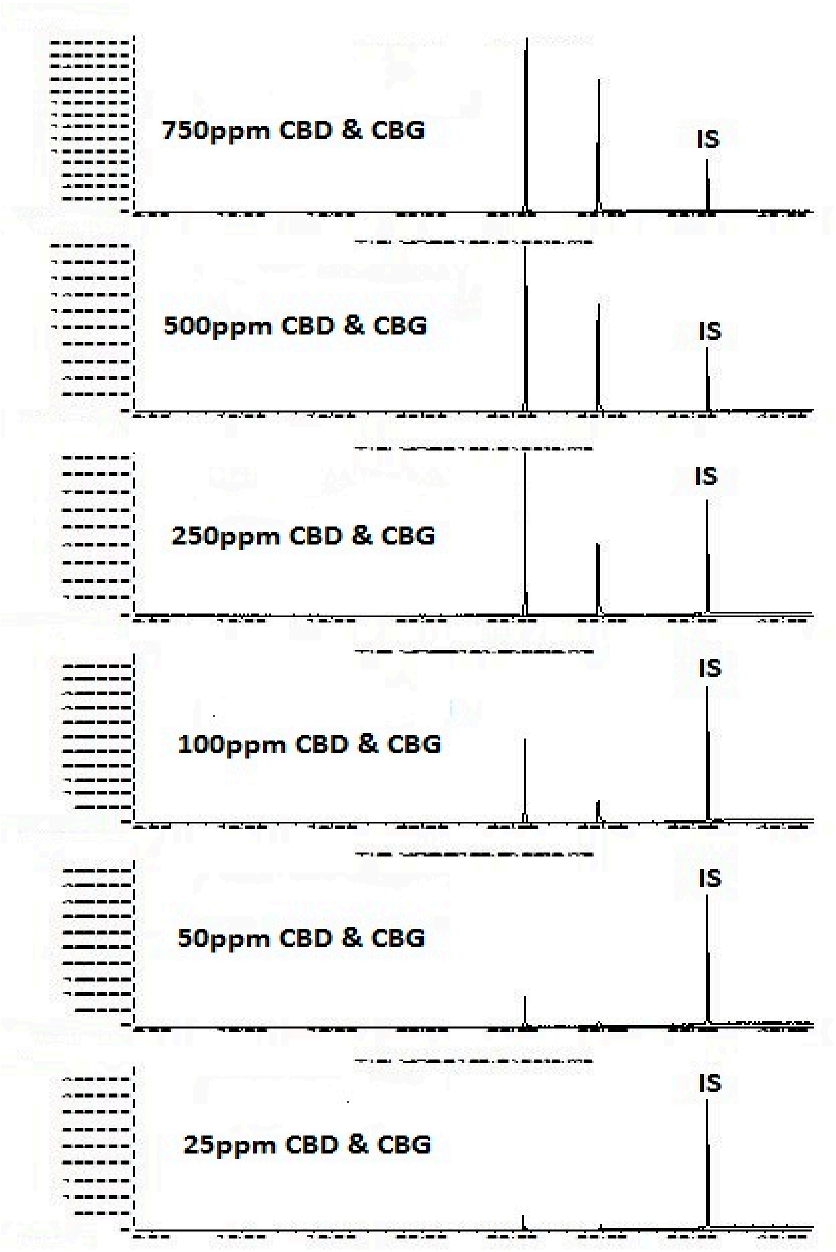

Figure S24: GC-MS chromatograms of CBD and CBG in different concentrations

Table S2: Comparison of a carboxylated cannabis extract with different analytical methods

	CBD %	CBG %	CBN %
COSY-NMR	37.6	2.34	ND
H-NMR	36.8	2.42	ND
GC-MS	35.5	2.38	-
HPLC	35.1	2.43	0.02
**% Relative difference COSY-NMR/H-NMR**	**2.13**	**3.42**	-
**% Relative difference COSY-NMR/GC-MS**	**5.92**	**1.71**	-
**% Relative difference COSY-NMR/HPLC**	**7.12**	**3.85**	-
**% Relative difference H-NMR/GC-MS**	**3.66**	**1.68**	-
**% Relative difference H-NMR/HPLC**	**4.84**	**0.41**	-

4. Method validation Tables

Table S3: LOD, LOQ, intra-day precision, inter-day precision and accuracy of ^{1}H qNMR quantitation method

H-NMR							
	CBD	CBDA	CBG	CBGA	CBN	Δ9-THCA	Δ9-THC
Proton	H-10 trans	H-10 cis	H-1′a/H-1′b	H-1′a/H-1′b	H-10	H-10a	H-10a
LOD (mg)	0.018	0.018	0.020	0.020	0.015	0.042	0.042
LOQ (mg)	0.060	0.060	0.066	0.066	0.050	0.930	0.930
Intra –day precision (%)	2.4	2.9	2.6	2.1	2.7	2.9	2.2
Inter-day precision (%)	2.9	3.2	3.1	2.9	3.1	3.3	3
Accuracy (%)	0.3	0.8	0.4	1.2	1.1	0.9	0.6

Table S4: LOD, LOQ intra-day precision, inter-day precision and accuracy of ^{1}H-^{1}H COSY NMR quantitation method

COSY-NMR					
	CBD	CBDA	CBG & CBGA	CBN	Δ9-THCA & Δ9-THC
Correlations (H-H)	H-10 trans / H-9	H-10 trans / H-10 cis	H-6′ / H-1′	H-10 / H-8	H-10a / H – 6a
LOD (mg)	0.11	0.12	0.1	0.08	0.08
LOQ (mg)	0.2	0.22	0.16	0.17	0.17

Intra –day precision (%)	2.6	5.3	5.4	4.3	2.9
Inter-day Precision (%)	4.7	6.3	4.2	5.6	3.1
Accuracy (%)	1.8	3.2	4.5	2.8	2.1

Table S5: Comparison of ^{1}H-qNMR and ^{1}H-^{1}H-COSY qNMR methods

	**Sample**	**CBD**	**CBD**	**CBDA**	**CBDA**	**CBD&CBDA**	**CBG&CBGA**	**CBG&CBGA**
		**(I)**	**%**	**(I)**	**%**	**%**	**(I)**	**%**
**^{1}H-NMR**	R1N135 (1)	0.33	0.28	4.88	4.72	5.00	0.75	0.73
	R1N135 (2)	0.33	0.28	4.91	4.75	5.03	0.78	0.76
	R1N135 (3)	0.34	0.29	4.94	4.78	5.07	0.77	0.75
	**R1N135 (Average)**	**0.33**	**0.28**	**4.91**	**4.75**	**5.03**	**0.77**	**0.75**
**H-NMR % RSD**			2.04		0.63	0.69		2.05
**COSY-NMR**	R1N135 (1)	2.22	0.25	27.54	4.69	4.93	88.95	0.72
	R1N135 (2)	2.32	0.25	28.94	4.92	5.18	87.71	0.71
	R1N135 (3)	2.44	0.26	29.47	5.01	5.28	89.57	0.72
	**R1N135 (Average)**	**2.33**	**0.26**	**28.65**	**4.87**	**5.13**	**88.74**	**0.71**
**COSY-NMR % RSD**			2.28		3.38	3.51		0.80
**% Relative difference**			**7.69**		**2.52**			**5.63**
**^{1}H-NMR**	R4N120 (1)	0.21	0.18	3.74	3.62	3.82	0.27	0.26
	R4N120 (2)	0.21	0.18	3.76	3.64	3.84	0.28	0.27
	R4N120 (3)	0.22	0.19	3.78	3.66	3.87	0.28	0.27
	**R4N120 (Average)**	**0.21**	**0.18**	**3.77**	**3.65**	**3.86**	**0.28**	**0.27**
**H-NMR % RSD**			3.15		0.55	0.65		2.16
**COSY-NMR**	R4N120 (1)	ND	ND	21.28	3.62	3.6	30.38	0.26
	R4N120 (2)	ND	ND	21.72	3.70	3.75	31.51	0.27
	R4N120 (3)	ND	ND	21.86	3.72	3.69	31.24	0.27
	**R4N120 (Average)**	**ND**	**ND**	**21.62**	**3.68**	**3.68**	**31.04**	**0.26**
**COSY-NMR % RSD**					1.44	2.05		2.16
**% Relative difference**					**0.82**	**4.97**		**3.84**
**^{1}H-NMR**	R3N110 (1)	ND	ND	ND	ND	ND	3.27	3.18
	R3N110 (2)	ND	ND	ND	ND	ND	3.28	3.19
	R3N110 (3)	ND	ND	ND	ND	ND	3.28	3.19
	R3N110 (Average)	ND	ND	ND	ND	ND	**3.28**	**3.19**
**H-NMR % RSD**								0.18
**COSY-NMR**	R3N110 (1)	ND	ND	ND	ND	ND	345.6	3.00
	R3N110 (2)	ND	ND	ND	ND	ND	346.9	3.01
	R3N110 (3)	ND	ND	ND	ND	ND	349.3	3.03

	R3N110 (Average)	ND	ND	ND	ND	ND	**347.3**	**3.01**
**COSY-NMR % RSD**								0.51
**% Relative difference**								**5.98**

4. ^{1}H-NMR and COSY- NMR experiment parameters.

4.1. ^{1}H-NMR experiment parameters

Table S6: ^{1}H-NMR acquisition parameters

**Acquisition Parameters**	**F1**
**PULPROG**	**zg**
**TD**	**32768**
**NS**	**16**
**DS**	**2**
**SW**	**13.155 ppm**
**SWH**	**5263.158 Hz**
**FIDRES**	**0.321238 Hz**
**FW**	**240000000.00 Hz**
**AQ**	**3.1129601 sec**
**RG**	**101**
**DW**	**95.000 usec**
**DECIM**	**24**
**DR**	**32**
**DDR**	**2**
**DE**	**6.50 μsec**
**QNP**	**1**
**NUC1**	**1H**
**O1**	**2400.78 Hz**

**SFO1**	**400.1324008 MHz**
**O1P**	**6000 ppm**
**BF1**	**400.1300000 MHz**
**LOCNUC**	**2H**
**SOLVENT**	**$CDCl_3$**
**PROBHD**	**11:5mmMultinucl.**
**EXP**	**PS done**
**RO**	**20 Hz**
**MASR**	**4200**
**NBL**	**1**
**TE**	**300.0 K**
**D1**	**10 sec**
**P0**	**10 usec**
**V9**	**5.00 %**
**WBST**	**1024**

4.2. COSY-NMR experiment parameters

Table S7: COSY-NMR acquisition parameters

**Acquisition Parameters**	**F2**	**F1**
**PULPROG**	**cosygpmfppf**	**-**
**TD**	**2048**	**256**
**NS**	**2**	**-**
**DS**	**16**	**-**
**ND0**	**-**	**1**

**IN0**	**-**	**0.00020600 sec**
**SW**	**12.1319 ppm**	**12.1319 ppm**
**SWH**	**4854.369 Hz**	**4854.360 Hz**
**FIDRES**	**5.086263 Hz**	**18.962343 Hz**
**FW**	**240000000.00 Hz**	**-**
**AQ**	**0.2109440 sec**	**-**
**RG**	**128**	**-**
**DW**	**96.000 usec**	**-**
**DWOV**	**4.000 usec**	**-**
**DECIM**	**24**	**-**
**DR**	**18**	**-**
**DDR**	**2**	**-**
**DE**	**8.00 usec**	**-**
**QNP**	**1**	**-**
**NUC1**	**1H**	**1H**
**O1**	**2400.78 Hz**	**2400.78 Hz -**
**SFO1**	**400.1324008 MHz**	**400.1324008 MHz**
**O1P**	**6000 ppm**	**6000 ppm**
**BF1**	**400.1300000 MHz**	**400.1300000 MHz**
**GRDPROG**	**1squa**	**-**
**LOCNUC**	**2H**	**-**
**SOLVENT**	**CDCl3**	**-**
**PROBHD**	**11:5mmMultinucl.**	**-**

**EXP**	**PS done**	**-**
**RO**	**20 Hz**	**-**
**MASR**	**4200**	**-**
**NBL**	**1**	**-**
**TE**	**300.0 K**	**-**
**D1**	**1.5 sec**	
**P0**	**10 usec**	
**V9**	**5.00 %**	**-**
**WBST**	**1024**	**-**
**WBSM**	**4.0000000 MHz**	**-**
